# Recruitment rates and strategies in exercise trials in cancer survivorship: a systematic review

**DOI:** 10.1007/s11764-023-01363-8

**Published:** 2023-04-06

**Authors:** Sophie A. Reynolds, Louise O’Connor, Anna McGee, Anna Quinn Kilcoyne, Archie Connolly, David Mockler, Emer Guinan, Linda O’Neill

**Affiliations:** 1 School of Medicine, Trinity College Dublin, University of Dublin, Dublin, Ireland; 2 Trinity St James’s Cancer Institute, Dublin, Ireland; 3 Discipline of Physiotherapy, School of Medicine, Trinity College Dublin, University of Dublin, Dublin, Ireland; 4 John Stearne Library, Trinity Centre for Health Sciences, St James’s Hospital, Dublin, Ireland

**Keywords:** Recruitment rate, Recruitment strategies, Cancer survivors, Exercise

## Abstract

**Purpose:**

Despite clear evidence-based supporting a benefit to exercise on physical and psychological metrics in patients with cancer, recruitment to exercise trials amongst cancer survivors is suboptimal. We explore current recruitment rates, strategies, and common barriers to participation in exercise oncology trials in cancer survivorship.

**Methods:**

A systematic review was conducted using a pre-defined search strategy in EMBASE, CINAHL, Medline, Cochrane Library, and Web of Science. The search was performed up to 28/02/2022. Screening of titles and abstracts, full-text review, and data extraction was completed in duplicate.

**Results:**

Of the 3204 identified studies, 87 papers corresponding to 86 trials were included. Recruitment rates were highly variable with a median rate of 38% (range 0.52–100%). Trials recruiting prostate cancer patients only had the highest median recruitment rate (45.9%) vs trials recruiting colorectal cancer patients only which had the lowest (31.25%). Active recruitment strategies such as direct recruitment via a healthcare professional were associated with higher recruitment rates (rho = 0.201, *p* = 0.064). Common reasons for non-participation included lack of interest (46.51%, *n* (number of studies) = 40); distance and transport (45.3%, *n* = 39); and failure to contact (44.2%, *n* = 38).

**Conclusions:**

Recruitment of cancer survivors to exercise interventions is suboptimal with barriers being predominantly patient-oriented. This paper provides the benchmark for current recruitment rates to exercise oncology trials, providing data for trialists planning future trial design and implementation, optimise future recruitment strategies, and evaluate their own recruitment success against current practice.

**Implications for Cancer Survivors:**

Enhanced recruitment to cancer survivorship exercise trials is necessary in facilitating the publication of definitive exercise guidelines, generalisable to varying cancer cohorts.

**PROSPERO registration number:**

CRD42020185968.

**Supplementary Information:**

The online version contains supplementary material available at 10.1007/s11764-023-01363-8.

## Background

Implementation of evidence-based healthcare is dependent upon stringent high-quality evidence from trials of robust methodological design, with inclusivity of representative patients, sufficient numbers to meet power calculations, and avoidance of bias. Large sample sizes are often required to minimise the risk of bias [1–3], produce data with narrow confidence intervals [4, 5], and enhance the statistical power [4–6] of a trial. The provision of such high-quality data may in turn underpin grade A recommendations that inform clinical practice [7, 8] and guidelines. Adequately powered trials assist with determining intervention efficacy, and external validity of results can be evaluated to a greater degree of certainty and guide the provision of evidence-based healthcare. Notwithstanding such major evident benefit of clinical trials, a bugbear of research is suboptimal recruitment of patients, with recruitment failure leading to the early termination of 19–40% of clinical trials [9]. Poor recruitment feeds into underpowered data and potential errors of interpretation with attendant risks of type II errors, wherein a null hypothesis is incorrectly accepted [6, 10]. These errors may lead to an over-exaggeration of treatment effect and fail to adequately assess associations with confounders that may impact intervention uptake [4, 11]. Moreover, a failure of trials to reach accrual targets results in increased costs and wasted resources [12, 13] and a failure of the fundamental objective to provide definitive conclusions informing practice [14]. Hence, trial recruitment represents a significant area of interest, in particular to ascertain the barriers and pitfalls that may be addressed.

An area of cancer research which has witnessed exponential growth in the past decade is that of exercise trials in cancer survivorship [14, 15]. The number of cancer survivors continues to increase globally due to advances in treatment options and increased early detection of cancers. In the USA alone, the American Cancer Society documented 16.9 million cancer survivors as of January 1st, 2019 [16]. Moreover, in the European context, ambitions exist to increase cancer survival beyond 10 years to 70% by 2035 [17]. However, whilst this trend is positive, there are now more and more people living with the significant health, social, and economic consequences of cancer and its treatments. The combined action of age-related physiological processes and pathological side effects related to cancer and its therapies has been shown to impact the long-term physical and psychological well-being of cancer survivors [18, 19]. These long-term effects including, but not limited to, cardiovascular disease [20–23], impaired physical function [24, 25], fatigue [26–31], and overall reduced quality of life [32–34] have prompted the movement towards the development of rehabilitative programmes in cancer survivorship [35–38] in order to enhance management of such symptoms and improve the overall quality of survivorship.

Exercise is a core rehabilitative intervention which has been utilised to varying levels of success to mitigate some of these negative sequelae of cancer and its treatments. As highlighted in the 2018 American College of Sports Medicine (ASCM) roundtable reports [14, 15], exercise has been shown to be a safe intervention for cancer survivors with strong evidence supporting its role in counteracting such cancer-related side effects as anxiety and depressive symptoms, fatigue, physical function, health-related quality of life, and lymphedema. With similar findings replicated following recent systematic review, the American Society of Clinical Oncology (ASCO) expert panel recommends that oncology healthcare providers endorse regular exercise by those patients undergoing active treatment [39]. Despite this, a number of knowledge gaps and biases still exist. There is, for instance, a predominance of exercise studies in cancer survivorship on common cancer types, namely, breast, prostate, and colorectal, with less on cancers such as lung, pancreas, and oesophageal [14, 15, 40], which can have a more significant physical toll. There is also arguably a bias towards inclusion of survivors with low or no disease burden and those with higher education status [14, 41, 42]. Accordingly, research conclusions may not be generalisable, and the direct application of current guidelines to alternate groups may not be valid [41]. A general gap also exist on the role of exercise on sleep [43–46], cognitive function [47, 48], pain [14, 49], and treatment tolerance [14, 50–52], in cancer patients. Importantly, optimal dosing for specific cancer types and patient sub-groups is absent, as trials directly comparing differing intervention prescriptions is minimal [14, 53]. Given this, the ASCM has suggested that future studies of adequate power and randomised controlled design be implemented in a variety of patient sub-groups, of varying prognoses, as to guide the development of exercise prescription in a manner similar to that seen in precision medicine [14].

A fundamental requirement for exercise trials in cancer survivorship is adequate recruitment. There has been an evident expansion of research in this field; however, recruitment of cancer patients to clinical trials has been shown to be less than 5% [54–56] with as little as 5.7% of these taking part in exercise interventions [57]. Continuing exercise oncology research in this way will waste valuable resources and slow the development and application of evidence-based rehabilitation for cancer survivors. As such, there is a clear need to gain greater insight into current recruitment practices used in exercise trials in cancer survivorship and to identify key barriers to participation for this specific cohort. As such, the aims of this systematic review are as follows:

i) Examine current recruitment rates to exercise trials amongst cancer survivors.
ii) Identify current recruitment strategies.
iii) Identify common barriers to participation in exercise trials in cancer survivorship.

## Methods

A systematic approach based on the PRISMA guidelines was applied in the reporting of this review. EMBASE, MEDLINE, CINAHL, Cochrane Library, and Web of Science were searched up until 28/02/2022. A search strategy was generated by the subject librarian (DM) using all keywords and subject headings included (Supplementary Material 1).

Eligibility criteria were (i) included adult cancer survivors, i.e. adult cancer patients with a histologically confirmed diagnosis of cancer of any type, who had completed primary treatment, e.g. surgery, neoadjuvant/adjuvant chemotherapy and/or radiotherapy; (ii) included a prescriptive exercise intervention; (iii) included a randomised controlled study design; (iv) included information regarding recruitment methods; and (vi) included information regarding recruitment rates. Exclusion criteria were (i) articles unavailable as full-text; (ii) articles unavailable in English; (iii) systematic reviews, meta-analysis, abstracts, conference proceedings, case studies, and letters to the editor; (iv) secondary articles, unless the original article failed to provide sufficient data (v) including participants under the age of 18 years of age; (vi) studies wherein recruitment rate is not provided nor calculable; and (vii) non-prescriptive exercise interventions or physical activity recommendations.

For the purpose of this review exercise was defined as “planned, structured and repetitive bodily movement, the objective of which is to improve or maintain physical fitness” [58]. This definition was inclusive of various exercise interventions including aerobic and resistance training, flexibility training such as yoga, and other physical activity programmes which followed a defined exercise prescription based on the frequency, intensity, type, and time (FITT) principle. Recruitment rate was defined as the percentage of potentially eligible participants that were recruited.

Utilising Covidence, an electronic systematic review management system, all titles and abstracts were reviewed by at least two independent reviewers from the review team (SR/LON/AM/AQK/AC). Articles not meeting the pre-defined inclusion criteria were excluded, with discrepancies resolved by a third independent reviewer (EG). The same process was applied to the subsequent full-text review. Details pertaining to author, year of publication, patient characteristics, recruitment rate, recruitment strategies, reasons for non-participation, and exercise interventions were extracted independently by SR, LON, LOC, AMcG, AQK, and AC. Where within text information regarding recruitment rate was insufficient, recruitment rates were calculated from information provided in the trials’ CONSORT flow diagram. Data was compared and inconsistencies resolved. Following extraction of recruitment methods and non-participation, findings were grouped according to common themes, with frequency expressed as a percentage of overall trials. In addition, the relationship between recruitment rates and method of recruitment was explored using Spearman rank order correlation in IBM SPSS. Assessment of risk of bias was not applicable to this systematic review as studies included were neither randomised nor blinded for the outcomes related to recruitment.

## Results

**Fig. 1:**
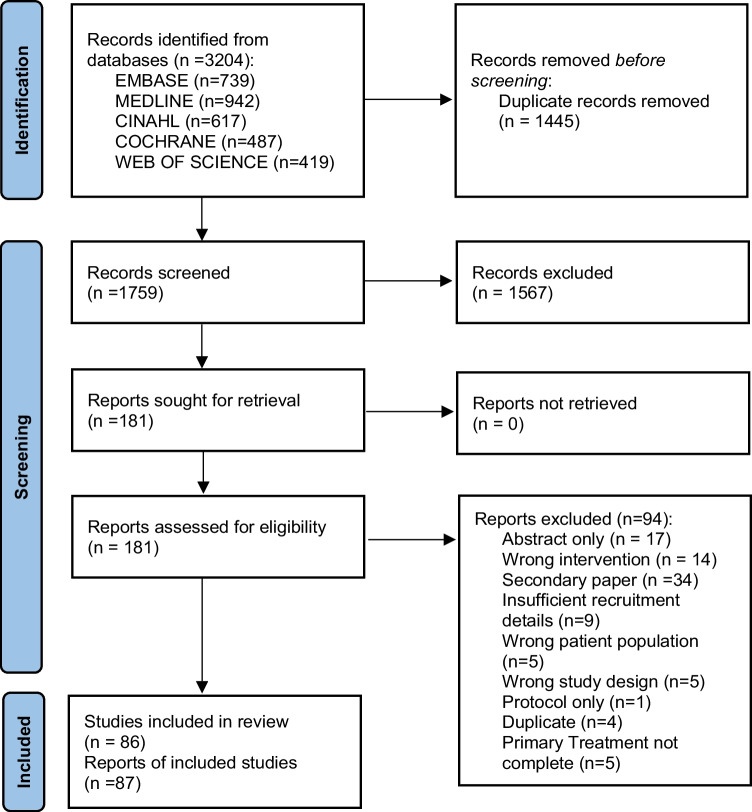
Selection process for eligible articles

The literature search results are presented in Fig. 1. A total of 3204 studies were identified through the pre-defined search strategy completed on February 28th, 2022. Following removal of duplicates, a total of 1759 citations underwent abstract and title screening. One hundred eighty-one papers underwent full-text screening, of which 87 papers accounting for 86 trials were included in the final review. The full list and description of included studies are included as Supplemental Material 2. The full reference list and reason for exclusion of reports at full-text review stage are provided in Supplemental Material 3. Trials were published between 2003 and 2021. Over two thirds of trials were conducted in North America (*n* = 60, 69.77%) with the remainder from Europe (*n* = 9, 10.47%), Australia and New Zealand (*n* = 6, 6.98%), the UK and Northern Ireland (*n* = 5, 5.81%), Asia (*n* = 5, 5.81%), and South America (*n* = 1, 1.16%). Breast cancer survivors were the most commonly studied cohort, being the most prominent cancer type in 50 trials (58.1%), 36 including breast cancer only and 14 trials where breast cancer accounted for over 50% of study participants. This was followed by prostate cancer (*n* = 9, 10.5%), colorectal (*n* = 6, 6.98%), and gynaecological (*n* = 3, 3.5%), and 31 trials included a mix of cancer types (36.04%). Median intervention duration was 5 months (range 0.25–24), with a median follow-up of 6 months (range 0.25–24). Combined aerobic and resistance training accounted for over a third of interventions (*n* = 31, 36.05%), followed by physical activity (*n* = 18, 20.93%). Other interventions included aerobic training alone (*n* = 13, 15.17%), resistance training alone (*n* = 11, 12.79%), yoga (*n* = 7, 8.14%), walking (*n* = 5, 5.81%), Qigong (*n* = 2, 2.33%), Tai Chi (*n* = 1, 1.16%), line dancing (*n* = 1, 1.16%), and wall-climbing (*n* = 1, 1.16%). Seven trials (8%) involved a combined exercise and dietary intervention (dietary advice and/or diet supplementation).

### Recruitment rates and methods to exercise trials for cancer survivors

Recruitment rates to individual trials are presented in Supplemental Material 2. The median recruitment rate for all trials was 38% (range 0.52–100%) (mean 42.96% (SD 27.2)). When categorised by cancer type (Table 1), trials recruiting prostate cancer patients only had the highest median recruitment rate of 45.9% (range 9.4–74%), followed by trials recruiting only breast cancer patients (median recruitment rate 44.4% (range 0.52–96.2%)), gynaecological cancer patients only (median recruitment rate 38.8% (range 21.1–90.91%)), trials with a mixed cancer population (median recruitment rate 36.6% (range 8–100%)), and those recruiting colorectal cancer patients only (median recruitment rate 31.25% (range 2.8–86.8%)).

**Table 1:** Median and mean recruitment rates per cancer type

Cancer type	Number of trials	Median (range) %	Mean (standard deviation) %
Breast	36	44.4 (0.52–96.2)	44.32 (27.29)
Colorectal	6	31.25 (2.8–86.8)	34.3 (29.67)
Gynaecological	3	38.8 (21.1–90.91)	50.27 (36.39)
Prostate	9	45.9 (9.4–74)	40.27 (20.43)
Mixed cancer cohort	31	36.6 (8–100)	43.29 (19.15)

Recruitment methods reported in included trials are described in Table 2 and include active recruitment strategies such as recruitment at medical or surgical clinics (*n* = 38, 44.2%), recruitment by healthcare professionals (*n* = 33, 38.4%), and advocacy groups or community events (*n* = 24, 27.9%) and passive recruitment strategies such as print materials or broadcast (*n* = 21, 24.4%), state cancer registries (*n* = 17, 19.8%), hospital (*n* = 16, 18.6%) or previous trial registries (*n* = 12, 13.7%), in-hospital advertisements (*n* = 10, 11.6%) and web-based advertisements (*n* = 10, 11.6%), and patient-led recruitment such as self-referral (*n* = 8, 9.3%) and word of mouth (*n* = 7, 8.1%). Correlation analysis showed that direct recruitment by medical professionals had a small but non-significant positive correlation with recruitment rate achieved (rho = 0.201, *p* = 0.064). Passive recruitment strategies such as invitation through state registries (rho = − 0.367, *p* = 0.001) and hospital registries (rho = − 0.255, *p* = 0.018) were negatively correlated with recruitment rates. No other significant correlations between recruitment method and rate were observed.

**Table 2:** Recruitment methods

Recruitment method	Description	Number of trials (%)
Active recruitment strategies		
Clinic(s)	Participant approached at oncology clinics attending for medical or surgical review, cancer treatment, or those identified following screening of clinic list(s)	38 (44.19)
Medical professional	Referral from physician, oncologist, or other treating medical professional involved in patient cancer care, either within the treating cancer facility or community-based	33 (38.37)
Advocacy groups/community events	The use of advocacy group events or trial promotion at community events wherein potential participants are likely to attend, i.e. health fairs, community organisation meetings	24 (27.91)
Passive recruitment strategies		
Print/broadcast advertisement	Newspapers, media announcements, social media, or flyers distributed within the community, i.e. gyms or workplaces	21 (24.42)
State registry	Patients invited to participate after being identified in state-wide or regional cancer database(s)	17 (19.77)
Hospital registry	Active and former cancer patients identified through hospital database(s)	16 (18.60)
Cancer trial registry	Former cancer trial participants consented for future contact	12 (13.95)
Web-based advertisement	Online advertisements to cancer forums, trial websites, or additional web-based platforms not otherwise specified	10 (11.63)
In-hospital advertisement	Distribution of placement of flyers, posters, or brochures at hospital facilities, outpatient clinics, or information centres	10 (11.63)
Patient-led recruitment		
Self-referral	Self- referral by participant to trial	8 (9.30)
Word of mouth	Potential participants informed of trial by active participants	7 (8.14)

### Reasons for non-participation in exercise trials for cancer survivors

Reasons for non-participation in exercise trials in cancer survivorship are outlined in Table 3. Over three quarters of trials failed to report specific reasons for non-participation (*n* = 66). The most common reasons for refusal to trial participation were distance and transport (*n* = 39, 45.35%), lack of interest (*n* = 40, 46.51%), failure to contact or no response (*n* = 38, 44.19%), time commitments (*n* = 35, 40.70%), and health concerns (*n* = 30, 34.88%). Other reasons were as follows: other commitments (*n* = 17, 19.77%), lost to follow-up (*n* = 13, 15.12%), already exercising (*n* = 13, 15.12%), not willing to be randomised (*n* = 10, 11.63%), and not willing to complete outcome measures (*n* = 5, 5.81%).

**Table 3:** Reasons for non-participation in trials

Reason for non-participation	Number of trials (%)
Decline, vague reason, or no reason provided	66 (76.74)
Not interested	40 (46.51)
Distance from research facility or insufficient transport available	39 (45.35)
Unable to contact or did not respond	38 (44.19)
Time commitment or too busy	35 (40.70)
Health concerns or felt too unwell	30 (34.88)
Other commitments, e.g. work, family	17 (19.77)
Already exercising	13 (15.12)
Lost to follow-up	13 (15.12)
Not willing to be randomised	10 (11.63)
Not willing to complete outcome measure(s)	5 (5.81)

## Discussion

This is the first systematic review, to our knowledge, that provides a comprehensive overview of recruitment practices used in the accrual of cancer survivors to exercise interventions. Our findings reveal that recruitment rates to exercise interventional trials amongst cancer survivors are suboptimal (median 38%) in contrast to cancer clinical trials wherein a recruitment rate of 55% [59] is considered typical and highlight that current recruitment strategies are of varying efficacy.

This review reinforces previous findings on exercise rehabilitation in specific cancer types. Two recent systematic reviews examining the safety and feasibility of exercise amongst colorectal cancer patients [60] and stage II + breast cancer survivors [61] reported recruitment rates of 38% (range 4–91%) and 56% (range 1–96%), respectively. For those with advanced disease, two independent reviews noted a 49% accrual rate to exercise interventions to be typical [62, 63], whilst in a review of interventions promoting physical activity amongst cancer survivors, Turner et al. [64] noted varying recruitment rates between 9.5 and 95%. Furthermore, this systematic review is also the first to provide a breakdown of recruitment rates per cancer type in cancer survivorship. Significant variance in recruitment rates were observed between trials of different cancer types, with prostate cancer trials recording a median recruitment rate of 45.9%, but colorectal cancer trials only reaching a median recruitment target of 31.25%. Similar variability in recruitment rates is observed in exercise trials in non-cancer chronic diseases, including chronic obstructive pulmonary disease [65] and cardiovascular disease [66–68], with recruitment to pulmonary and cardiac rehabilitation varying from 35 to 100% and 4 to 100%, respectively. Accordingly, findings to date demonstrate an inherent variance in the degree of participation in exercise trials throughout healthcare but with the majority of studies recruiting less than 50%.

A further dimension of the recruitment problem is generalisability of data and results within a disease population [69–71]. For instance, the exclusion of ethnic and racial minorities [72–74], socioeconomically disadvantaged, and less educated cohorts [75–77] has increased over the last decade with studies demonstrating a disproportionate lack of awareness of clinical trial availability, despite equivalent willingness to participate [72, 78]. Patient barriers [72, 79, 80], trial design [79–82], and recruiter bias [79, 83] are amongst a few established factors limiting the inclusion of such patient groups. Similarly, as highlighted in this review, there is a marked predominance of specific cancer types, with 86.05% of included studies exclusively encompassing breast, prostate, and colorectal cancers, and moreover particularly focused on early-stage cancer survivors. These methodological errors may limit the external validity and broad generalisability, for instance, to other cancer types, patients with more advanced disease and particular social and racial demographics. A reasonable interpretation of the review data is that the majority of research falls short of the ACSM projection for precision exercise prescription [14] which requires the adaption of such exercise-based clinical trials as to include such minority groups in order to facilitate the development of a body of research that allows for individualised exercise prescriptions for cancer survivors.

If appropriate representation within trials is a fundamental to published trials, a more fundamental problem, perhaps easily addressed, is the methodology to recruit patients and the barriers that exist that may be circumvented. In this review, we have highlighted that recruitment of cancer survivors to exercise interventions is largely medically based, with correlation analysis suggesting a small but non-significant effect through direct recruitment by healthcare professional (HCP), with the converse appearing true for passive methods including the use of institutional databases. Greater access to the intended trial cohort by the healthcare team, a greater proportion of research-orientated clinicians and healthcare professionals, and trust established through patient–clinician relationships provide a significant opportunity for improved recruitment [84, 85]. However, for patients, misunderstanding of RCT concepts and design, discomfort with RCT eligibility criteria, lack of equipoise between treatment options, and difficulty exploring patient preferences are amongst a few clinician-related barriers which may impact recruitment [81, 86]. Although training has shown to improve recruiter confidence and communication [87], effects on trial accrual and patient satisfaction have been suboptimal [86, 87]. An intriguing suggestion to improve medically based recruitment is a prospective ‘trial by design’ [88], in which recruitment practices are evaluated and altered during the trials active phase. The QuinteT Recruitment Intervention is a similar concept: a two-phase intervention evaluating trial practices through reviews of trial documentation, audio-recordings of recruitment sessions, and interviews with recruitment staff and declining participants, which in turn guide revision of recruitment practices [89]. Such methods allow for alterations of recruitment design which address site- and cohort-specific barriers. Reporting such alterations in line with embedded recruitment trial guidelines [90] provides opportunity for accurate documentation of changes in recruitment strategies and direct comparison on recruitment effects.

It is clear from this review that the greatest barrier to participation in exercise trials in cancer survivorship are patient-centric, with transport and distance from intervention site, disinterest, time, commitments, and health concerns being commonly cited, consistent with previous reports [91–96]. Attempts made to date to implement strategies to enhance recruitment to clinical trials have largely not met their intended outcomes [97]. The absence of clarity as to why recruitment failure remains so prominent prompted the Prioritising Recruitment in Randomized Trials Priority Setting Partnership (PRioRiTy PSP), a collaborative effort aimed at identifying current recruitment uncertainties and prioritising the most relevant unanswered questions regarding recruitment [98]. This was done by way of steering groups and workshops inclusive of all research stakeholders from patients, recruiters, and those with research expertise and ultimately highlighted the need for greater public and patient involvement (PPI), defined as “research being carried out ‘with’ or ‘by’ members of the public rather than ‘to’, ‘about’ or ‘for’ them” [99]. Consequently, PPI has become a central strategy in optimising recruitment to clinical trials [100–102], demonstrating modest but significant increases in recruitment rates when applied to areas of recruitment processes and trial design [103, 104]. Relating to cancer specifically, the use of PPI within clinical cancer trials has grown significantly, yet persistent issues regarding inclusivity of cohort representation remain [105]. Hence, the core problem that this initiative is designed to mitigate remains a concern. Furthermore, owing in part to poor standards of reporting, optimal PPI strategies are less clear [102, 106]. However, the recent expansion of study within a trial (SWAT) trials and the Trial Forge Initiative [107] within Ireland and the UK may aid in shedding some light on these current uncertainties and provide standardised, easily interpretable data which can guide future recruitment practices.

## Limitations of review

We acknowledge some limitations. Although efforts were made to ensure all relevant papers were included, with a review of references and an additional hand search, it is possible that studies were missed or not included due to their unavailability in full-text form or in the English language. Furthermore, due to the heterogeneity of recruitment approaches, our review is unable to provide clear insight into definitive and effective recruitment measures or suggest optimal recruitment methods for variable cancer survivorship cohorts. Future studies should comparatively examine varying recruitment methods for cohorts of different cancer types and stages, disease, and socioeconomic burden. We also acknowledge that only RCTs including participants who had completed primary treatment prior to participation in an exercise intervention were included in this review. As the uptake of exercise interventions during active treatment increases, it would be useful to conduct a subsequent review into recruitment rates and strategies in that population.

## Conclusion

Given the scientific and clinical evidence supporting the clear benefit of exercise on physical and mental well-being in cancer survivorship, recruitment strategies within such trials should be of a high priority in cancer research. This systematic review highlights that recruitment rates to exercise trials in cancer survivorship are most often suboptimal (median recruitment rate 38%), and greater utilisation of trials methodology research is required in such trials to improve patient recruitment and the quality of trials and the strength of the evidence and recommendations. The findings of this review provide trialists in exercise oncology research with a comprehensive overview of recruitment rates, strategies, and reasons for non-participation and should be invaluable in future planning of trials in this field.

## Supplementary Information

Below is the link to the electronic supplementary material.

Supplementary file1 (DOCX 19 KB)

Supplementary file2 (DOCX 52 KB)

Supplementary file3 (DOCX 44 KB)

## Data Availability

All data generated or analysed during this study are included in this published article and its supplementary information files.
