# Correction: Recruitment rates and strategies in exercise trials in cancer survivorship: a systematic review

**DOI:** 10.1007/s11764-024-01716-x

**Published:** 2024-11-18

**Authors:** Sophie A. Reynolds, Louise O’Connor, Anna McGee, Anna Quinn Kilcoyne, Archie Connolly, David Mockler, Emer Guinan, Linda O’Neill

**Affiliations:** 1 School of Medicine, Trinity College Dublin, University of Dublin, Dublin, Ireland; 2 Trinity St James’s Cancer Institute, Dublin, Ireland; 3 Discipline of Physiotherapy, School of Medicine, Trinity College Dublin, University of Dublin, Dublin, Ireland; 4 John Stearne Library, Trinity Centre for Health Sciences, St James’s Hospital, Dublin, Ireland

**Correction: J Cancer Surviv (2024) 18:1233–1242**

10.1007/s11764-023-01363-8

In the original paper of the article “Recruitment rates and strategies in exercise trials in cancer survivorship: A systematic review’’ published in J Cancer Surviv (2024) 18:1233–1242, the results paragraph “Reasons for non-participation in exercise trials for cancer survivors” was not updated to reflect the final results of the review which are presented in Table 3.

The authors apologize for this error and any confusion it may have caused.

The original article has been corrected.

