# Supplementary material for: Recruitment rates and strategies in exercise trials in cancer survivorship: a systematic review
Source: J Cancer Surviv. 2023 Apr 6;18(4):1233–42. doi: 10.1007/s11764-023-01363-8 (PMC11324688; doi:10.1007/s11764-023-01363-8)
Supplement: Supplementary file 1 — Supplementary file1 (DOCX 19 KB) [file 11764_2023_1363_MOESM1_ESM.docx]

**Supplemental Material 1 – Search Strategy**

**Title:** Recruitment Rates and Strategies in Exercise Trials in Cancer Survivorship - A Systematic Review

**Authors:** Sophie Reynolds^1,2^, Louise O’Connor^2,3^, Anna McGee^1,2^, Anna Quinn Kilcoyne^1,2^, Archie Connolly^1,2^, David Mockler^4^, Emer Guinan^1,2^, Linda O’Neill^2,3^

**Affiliations:**

^1^ School of Medicine, Trinity College Dublin, the University of Dublin, Dublin, Ireland

^2^ Trinity St James’s Cancer Institute, Dublin, Ireland

^3^ Discipline of Physiotherapy, School of Medicine, Trinity College Dublin, the University of Dublin, Dublin, Ireland

^4^ John Stearne Library, Trinity Centre for Health Sciences, St James’s Hospital, Dublin, Ireland

**Corresponding Author:**

Dr Linda O’Neill

Discipline of Physiotherapy,

Trinity Centre for Health Sciences, St James’s Hospital,

Dublin 8

Email: [oneilll8@tcd.ie](mailto:oneilll8@tcd.ie)

Telephone: +353 1 8964809

**Supplemental Material 1** – Search Strategy

| **Database** | **Search Strategy** |
| --- | --- |
| EMBASE | 1. 'cancer survivor'/exp OR 'cancer survival'/exp 2. Survivo?r*:ti,ab 3. #1 OR #2 4. 'patient selection'/exp OR 'patient compliance'/exp 5. ((recruit* OR enrol* OR retention OR retain* OR adhere* OR compliance) NEAR/5 (strateg* OR plan* OR program* OR barrier* OR facilitator* OR rate OR rate$)):ti,ab 6. ((recruit* OR enrol* OR retention OR retain*) NEAR/5 (survivor*)):ti,ab 7. ((Patient* OR criteria OR subject* OR volunteer* OR treatment*) NEAR/2 selection*):ti,ab 8. #4 OR #5 OR #6 OR #7 9. 'exercise'/exp OR 'kinesiotherapy'/exp OR 'physical activity'/exp OR 'physical activity, capacity and performance'/de OR 'training'/de OR 'endurance'/de OR 'exercise tolerance'/de OR 'physical capacity'/de OR 'sport'/exp 10. (strength* or isometric* or isotonic* or isokinetic* OR exercis*):ti,ab 11. (resistance NEAR/3 train*):ti,ab 12. ((physical* or motion* or cardiopulmonary or cardiorespiratory) NEAR/3 (fit* or therap*)):ti,ab 13. (treadmill* or cross-train* or rowing or sport* OR exercise* OR ‘physical activit*’ OR aerobic* OR run or jog* or running OR walk or walks or walking OR gym* OR yoga oR pilates OR ‘recreation* activit*’ OR zumba or salsa* OR cycling or bicycle or bike or swim* or dance or dancer* or dances or dancing):ti,ab 14. (circuit* NEAR/1 train*):ti,ab 15. (keep* NEAR/1 (active or fit)):ti,ab 16. #9 OR #10 OR #11 OR #12 OR #13 OR #14 OR #15 17. #3 AND #8 AND #16 18. 'conference abstract':it OR 'conference review':it OR 'editorial':it OR 'letter':it 19. #17 NOT #18 |
| MEDLINE | 1. Survivors/ OR Cancer Survivors/ 2. Survivo?r*.ti,ab. 3. or/1-2 4. exp Patient Selection/ OR exp Patient Compliance/ 5. ((recruit* OR enrol* OR retention OR retain* OR adhere* OR compliance) adj5 (strateg* OR plan* OR program* OR barrier* OR facilitator* OR rate OR rate$)).ti,ab . 6. ((recruit* OR enrol* OR retention OR retain*) adj5 (survivor*)).ti,ab. 7. ((Patient* OR criteria OR subject* OR volunteer* OR treatment*) adj2 selection*).ti,ab. 8. or/4-7 9. exp Exercise/ OR exp Exercise Therapy/ OR exp Physical Fitness/ OR exp "physical education and training"/ OR exp "Exercise Movement Techniques"/ or physical endurance/ or exercise tolerance/ OR Physical Exertion/ or exp Sports/ or Dancing/ 10. (strength* or isometric* or isotonic* or isokinetic* or exercis*).ti,ab. 11. (resistance adj3 train*).ti,ab. 12. ((physical* or motion* or cardiopulmonary or cardiorespiratory) adj3 (fit* or therap* or activit*)).ti,ab. 13. (treadmill* or cross-train* or rowing or sport* or exercise* or physical* activit* or aerobic* or run or jog* or running or walk or walks or walking or gym* or yoga or pilates or "recreation* activit*" or zumba or salsa* or cycling or bicycle or bike or swim* or dance or dancer* or dances or dancing or physiotherapy* or physical therap*).ti,ab. 14. (circuit* adj1 train*).ti,ab. 15. (keep* adj1 (active or fit)).ti,ab. 16. or/9-15 17. and/3,8,16 |
| CINAHL | 1. (MH "Cancer Survivors") OR (MH "Survivors") 2. TI (Survivor*) OR AB (Survivor*) 3. S1 OR S2 4. (MH "Patient Selection") OR (MH "Patient Compliance") 5. TI ((recruit* OR enrol* OR retention OR retain* OR adhere* OR compliance) N5 (strateg* OR plan* OR program* OR barrier* OR facilitator* OR rate OR rate$)) OR AB ((recruit* OR enrol* OR retention OR retain* OR adhere* OR compliance) N5 (strateg* OR plan* OR program* OR barrier* OR facilitator* OR rate OR rate$)) 6. TI ((recruit* OR enrol* OR retention OR retain*) N5 (survivor*)) OR AB ((recruit* OR enrol* OR retention OR retain*) N5 (survivor*)) 7. TI ((Patient* OR criteria OR subject* OR volunteer* OR treatment*) N2 selection*) OR AB ((Patient* OR criteria OR subject* OR volunteer* OR treatment*) N2 selection*) 8. S4 OR S5 OR S6 OR S7 9. (MH "Exercise+") OR (MH "Physical Activity") OR (MH "Physical Fitness+") OR (MH "Physical Performance") OR (MH "Sports+") OR (MH "Resistance Training") OR (MH "Therapeutic Exercise+") OR (MH "Exercise Intensity") 10. TI (strength* or isometric* or isotonic* or isokinetic* OR exercis*) OR AB (strength* or isometric* or isotonic* or isokinetic* OR exercis*) 11. TI (resistance N3 train*) OR AB (resistance N3 train*) 12. TI ((physical* or motion* or cardiopulmonary or cardiorespiratory) N3 (fit* or therap*)) OR AB ((physical* or motion* or cardiopulmonary or cardiorespiratory) N3 (fit* or therap*)) 13. TI (treadmill* or cross-train* or rowing or sport* OR exercise* OR "physical activit*" OR aerobic* OR run or jog* or running OR walk or walks or walking OR gym* OR yoga oR pilates OR "recreation* activit*" OR zumba or salsa* OR cycling or bicycle or bike or swim* or dance or dancer* or dances or dancing) OR AB (treadmill* or cross-train* or rowing or sport* OR exercise* OR "physical activit*" OR aerobic* OR run or jog* or running OR walk or walks or walking OR gym* OR yoga oR pilates OR "recreation* activit*" OR zumba or salsa* OR cycling or bicycle or bike or swim* or dance or dancer* or dances or dancing) 14. TI (circuit* N1 train*) OR AB (circuit* N1 train*) 15. TI (keep* N1 (active or fit)) OR AB (keep* N1 (active or fit)) 16. S9 OR S10 OR S11 OR S12 OR S13 OR S14 OR S15 17. S3 AND S8 AND S16 |
| Cochrane Library | 1. [mh “Survivors”] OR [mh “Cancer Survivors”] 2. Survivo?r*:ti,ab,kw 3. #1 OR #2 4. [mh “patient selection”] OR [mh “patient compliance”] 5. ((recruit* OR enrol* OR retention OR retain* OR adhere* OR compliance) NEAR/5 (strateg* OR plan* OR program* OR barrier* OR facilitator* OR rate OR rate$)):ti,ab,kw 6. ((recruit* OR enrol* OR retention OR retain*) NEAR/5 (survivor*)):ti,ab,kw 7. ((Patient* OR criteria OR subject* OR volunteer* OR treatment*) NEAR/2 selection*):ti,ab,kw 8. #4 OR #5 OR #6 OR #7 9. [mh “Exercise”] OR [mh “Exercise Therapy”] OR [mh “Physical Fitness”] OR [mh "physical education and training"] OR [mh "Exercise Movement Techniques"] or [mh “physical endurance”] or [mh “exercise tolerance”] OR [mh “Physical Exertion”] or [mh “Sports”] or [mh “Dancing”] 10. (strength* or isometric* or isotonic* or isokinetic* OR exercis*):ti,ab,kw 11. (resistance NEAR/3 train*):ti,ab,kw 12. ((physical* or motion* or cardiopulmonary or cardiorespiratory) NEAR/3 (fit* or therap*)):ti,ab,kw 13. (treadmill* or cross-train* or rowing or sport* OR exercise* OR "physical activit*" OR aerobic* OR run or jog* or running OR walk or walks or walking OR gym* OR yoga oR pilates OR "recreation* activit*" OR zumba or salsa* OR cycling or bicycle or bike or swim* or dance or dancer* or dances or dancing):ti,ab,kw 14. (circuit* NEAR/1 train*):ti,ab,kw 15. (keep* NEAR/1 (active or fit)):ti,ab,kw 16. #9 OR #10 OR #11 OR #12 OR #13 OR #14 OR #15 17. #3 AND #8 AND #16 18. 'conference abstract':it OR 'conference review':it OR ‘editorial’:it OR ‘letter’:it 19. #17 NOT #18 |
| Web of Science | 1. TI =((cancer NEAR/5 Survivor*) AND (((recruit* OR enrol* OR retention OR retain* OR adhere* OR compliance) NEAR/5 (strateg* OR plan* OR program* OR barrier* OR facilitator* OR rate OR rate*)) OR ((recruit* OR enrol* OR retention OR retain*) NEAR/5 (survivor*)) OR ((Patient* OR criteria OR subject* OR volunteer* OR treatment*) NEAR/2 selection*)) AND ((strength* or isometric* or isotonic* or isokinetic* OR exercis*) OR (resistance NEAR/3 train*) OR ((physical* or motion* or cardiopulmonary or cardiorespiratory) NEAR/3 (fit* or therap*)) OR (treadmill* or cross-train* or rowing or sport* OR exercise* OR "physical activit*" OR aerobic* OR run or jog* or running OR walk or walks or walking OR gym* OR yoga oR pilates OR "recreation* activit*" OR zumba or salsa* OR cycling or bicycle or bike or swim* or dance or dancer* or dances or dancing) OR (circuit* NEAR/1 train*) OR (keep* NEAR/1 (active or fit)))) 2. AB =((cancer NEAR/5 Survivor*) AND (((recruit* OR enrol* OR retention OR retain* OR adhere* OR compliance) NEAR/5 (strateg* OR plan* OR program* OR barrier* OR facilitator* OR rate OR rate*)) OR ((recruit* OR enrol* OR retention OR retain*) NEAR/5 (survivor*)) OR ((Patient* OR criteria OR subject* OR volunteer* OR treatment*) NEAR/2 selection*)) AND ((strength* or isometric* or isotonic* or isokinetic* OR exercis*) OR (resistance NEAR/3 train*) OR ((physical* or motion* or cardiopulmonary or cardiorespiratory) NEAR/3 (fit* or therap*)) OR (treadmill* or cross-train* or rowing or sport* OR exercise* OR "physical activit*" OR aerobic* OR run or jog* or running OR walk or walks or walking OR gym* OR yoga oR pilates OR "recreation* activit*" OR zumba or salsa* OR cycling or bicycle or bike or swim* or dance or dancer* or dances or dancing) OR (circuit* NEAR/1 train*) OR (keep* NEAR/1 (active or fit)))) 3. #1 OR #2 |
