# Supplementary material for: Recruitment rates and strategies in exercise trials in cancer survivorship: a systematic review
Source: J Cancer Surviv. 2023 Apr 6;18(4):1233–42. doi: 10.1007/s11764-023-01363-8 (PMC11324688; doi:10.1007/s11764-023-01363-8)
Supplement: Supplementary file 2 — Supplementary file2 (DOCX 52 KB) [file 11764_2023_1363_MOESM2_ESM.docx]

**Supplemental Material 2 –** Details of Individual Studies by Cancer Type

**Title:** Recruitment Rates and Strategies in Exercise Trials in Cancer Survivorship - A Systematic Review

**Authors:** Sophie Reynolds^1,2^, Louise O’Connor^2,3^, Anna McGee^1,2^, Anna Quinn Kilcoyne^1,2^, Archie Connolly^1,2^, David Mockler^4^, Emer Guinan^1,2^, Linda O’Neill^2,3^

**Affiliations:**

^1^ School of Medicine, Trinity College Dublin, the University of Dublin, Dublin, Ireland

^2^ Trinity St James’s Cancer Institute, Dublin, Ireland

^3^ Discipline of Physiotherapy, School of Medicine, Trinity College Dublin, the University of Dublin, Dublin, Ireland

^4^ John Stearne Library, Trinity Centre for Health Sciences, St James’s Hospital, Dublin, Ireland

**Corresponding Author:**

Dr Linda O’Neill

Discipline of Physiotherapy,

Trinity Centre for Health Sciences, St James’s Hospital,

Dublin 8

Email: [oneilll8@tcd.ie](mailto:oneilll8@tcd.ie)

Telephone: +353 1 8964809

**Supplemental Material 2 –** Details of Individual Studies by Cancer Type

**Acute Myeloid Leukemia**

| **Author (Country)** | **Sample Size (n)** | **Gender (F:M, %)** | **Cancer types** | **Mean Age (Years)** | **Exercise Intervention(s)** | **Recruitment Rate (%)** |
| --- | --- | --- | --- | --- | --- | --- |
| Alibhai et al (Canada) (1) | 38 | 55.3 : 44.7 | Acute Myeloid Leukaemia (100%) | 56.1 (SD 8.7) | *Intervention:* AET, RET & stretching  *Control:* Waitlist control | 38.5 |

**Breast Cancer**

| **Author (Country)** | **Sample Size (n)** | **Gender (F:M, %)** | **Cancer types** | **Mean Age (Years)** | **Exercise Intervention(s)** | **Recruitment Rate (%)** |
| --- | --- | --- | --- | --- | --- | --- |
| Anderson et al (USA) (2) | 104 | 100 : 0 | Breast (100%) | 53.6 (range 32-82) | *Intervention:* AET & RET  *Control:* Usual care | 25 |
| Artese et al (USA) (3) | 44 | 100:0 | Breast (100%) | 60.3 (SD 8.3) | *Intervention:* Functional Impact Training  *Control:* Yin Yoga | 50 |
| Cohen et al (USA, Canada) (4) | 40 | 100 : 0 | Breast (100%) | 57.33 (SD 8.75) | *Intervention:* combined AET with guided-mindfulness relaxation *or* AET only *or* relaxation only | 81.63 |
| DeMello et al (USA) (5) | 76 | 100 : 0 | Breast (100%) | 55.62 (SD 9.55) | *Intervention:* Physical activity & peer-based support  *Control:* Peer-based support only | 33 |
| Dieli-Conwright et al (USA) (6) | 100 | 100 : 0 | Breast (100%) | 53 (SD 10.4) | *Intervention:* AET & RET  *Control:* Usual care & daily accelerometer use | 37.5 |
| Dos Santos et al (Brazil) (7) | 26 | 100 : 0 | Breast (100%) | Intervention: 55.0 (SD 5.8)  Control: 54.3 (SD 5.2) | *Intervention:* RET  *Control:* Usual care | 11.6 |
| Greenlee et al (USA) (8) | 42 | 100 : 0 | Breast (100%) | 50.7 (SD 8.9) | *Intervention:* Diet & circuit-based weight loss intervention  *Control:* Waitlist control | 45.7 |
| Irwin et al (USA) (9) | 75 | 100 : 0 | Breast (100%) | Intervention: 56.5 (SD 9.5)  Control: 55.1 (SD 7.7) | *Intervention:* AET  *Control:* Usual care | 13.1 |
| Irwin et al (USA) (10) | 121 | 100 : 0 | Breast (100%) | Intervention: 62.0 (SD 7.0)  Control: 60.5 (SD 7.0) | *Intervention:* AET & RET  *Control:* Usual care | 17.2 |
| Jones et al (New Zealand) (11) | 51 | 100 : 0 | Breast | Intervention: 55.8 (SD 7.2)  Control: 55.9 (SD 7.1) | *Intervention:* Circuit-based AET & RET  *Control:* Waitlist control | 96.2 |
| Kiecolt-Glaser et al (USA) (12) | 200 | 100 : 0 | Breast (100%) | 51.6 (SD 9.2) | *Intervention:* Hatha yoga  *Control:* Waitlist control | 70.2 |
| Kim et al (South Korea) (13) | 43 | 100 : 0 | Breast (100%) | Intervention: 55.7 (SD 5.3)  Control: 56.3 (SD 6.7) | *Intervention:* AET & RET with calcium & vitamin D supplements  *Control:* Supplements only | 36.8 |
| Kim et al (South Korea)(14) | 50 | 100:0 | Breast (100%) | 49.0 (SD 7.35) | *Intervention:* AET & RET  *Control:* Print material | 90 |
| Littman et al (USA) (15) | 63 | 100 : 0 | Breast (100%) | Intervention: 60.6 (SD 7.1)  Control: 58.2 (SD 8.8) | *Intervention:* Viniyoga  *Control:* Waitlist control | 76.8 |
| Loh et al (Malaysia) (16) | 197 | 100 : 0 | Breast (100%) | Not provided | *Intervention:* Qigong *or* group line-dancing  *Control:* Usual care | 18.5 |
| Matthews et al (USA) (17) | 36 | 100 : 0 | Breast (100%) | Intervention: 51.3 (SD 9.0)  Control: 56.9 (SD 12.3) | *Intervention:* Walking  *Control:* Waitlist control | 19.7 |
| McDonald et al (Australia) (18) | 49 | 100 : 0 | Breast (100%) | 48.9 (SD 1.4) | *Intervention:* Omega-3 supplements ± nutrition & PA education & circuit-based AET & RET  *Control: E*ducation with olive oil | 65.3 |
| Milne et al (Australia) (19) | 60 | 100 : 0 | Breast (100%) | 55.1 (SD 8.2) | *Intervention:* AET & RET  *Control:* Waitlist control | 53.7 |
| Owusu et al (USA) (20) | 213 | 100:0 | Breast (100%) | 70 (range 65-88) | *Intervention:* moderate-intensity AET & RET group training program  *Control:* Health education & support groups | 4.4 |
| Pinto et al (USA) (21) | 86 | 100 : 0 | Breast (100%) | 53.14 (SD 9.7) | *Intervention:* PA  *Control:* Contact control | 69.9 |
| Pinto et al (USA) (22) | 192 | 100 : 0 | Breast (100%) | 60.0 (SD 9.9) | *Intervention:* PA  *Control:* Contact control | 71 |
| Rogers et al (USA) (23) | 222 | 100 : 0 | Breast (100%) | 54.5 (SD 8.5) | *Intervention:* PA  *Control:* Usual care | 68.9 |
| Ruiz-Vozmediano et al (Spain) (24) | 72 | 100 : 0 | Breast (100%) | Intervention: 51.32 (SD 10.15)  Control: 48.33 (SD 7.72) | *Intervention:* PA  *Control:* Usual care | 80.9 |
| Sanft et al (USA) (25) | 151 | 100 : 0 | Breast (100%) | 57.8 (SD 7.7) | *Intervention:* PA  *Control:* Usual care | 29.3 |
| Schmitz et al (USA) (26) | 85 | 100 : 0 | Breast (100%) | 53 (range 34-75) | *Intervention:* RET  *Control:* Waitlist control | 34.8 |
| Schmitz et al (USA) (27) | 154 | 100 : 0 | Breast (100%) | Intervention: 54 (SD 8)  Control: 56 (SD 8) | *Intervention:* RET  *Control:* Non-exercising control | 8.5 |
| Sheppard et al (USA) (28) | 31 | 100 : 0 | Breast (100%) | 54.7 (SD 9.8) | *Intervention:* PA  *Control:* Health information | 46.3 |
| Stan et al (USA) (29) | 34 | 100 : 0 | Breast (100%) | 62.1 (SD 8.1) | *Intervention:* Yoga  *Control:* RET | 9.3 |
| Sturgeon et al (USA) (30) | 35 | 100 : 0 | Breast (100%) | 46.1 (SD 4) | *Intervention:* AET & RET  *Control:* Waitlist control | 63 |
| Taylor et al (USA) (31) | 33 | 100 : 0 | Breast (100%) | Intervention: 54.9 (SD 8.8)  Control: 52.6 (SD 8.2) | *Intervention:* Restorative yoga  *Control:* Waitlist control | 64.7 |
| Thomas et al (USA) (32) | 121 | 100 : 0 | Breast (100%) | 62.0 (SD 7) | *Intervention:* AET & RET  *Control:* Usual care | 16.5 |
| von Gruenigen et al (USA) (33) | 75 | 100 : 0 | Breast (100%) | Intervention: 57.0 (SD 8.65)  Control: 58.9 (SD 10.9) | *Intervention:* PA  *Control:* Usual care | 18.8 |
| Waltman et al (USA) (34) | 223 | 100 : 0 | Breast (100%) | 58.69 (SD 7.5) | *Intervention:* RET & bisphosphonate, calcium & vitamin D medication  *Control:* Medication only | 77.6 |
| Winkels et al (USA) (35)**^Data obtained from Sturgeon et al. (36)^* | 351 | 100 : 0 | Breast (100%) | 59.5 (SD 8.9) | *Intervention:* AET & RET ± weight-loss intervention *or* weight-loss only  *Control:* Usual care | 0.52 |
| Winters-Stone et al (USA) (37) | 71 | 100 : 0 | Breast (100%) | Intervention: 46.5 (SD 5.0)  Control: 46.4 (SD 4.9) | *Intervention:* RET & impact training  *Control:* Stretching | 46.1 |
| Winters-Stone et al (USA) (38) | 106 | 100 : 0 | Breast (100%) | *Intervention:* 62.3 (SD 6.7)  *Control:* 62.2 (SD 6.7) | *Intervention:* RET & impact training  *Control:* Stretching | 43.1 |

**Colorectal Cancer**

| **Author (Country)** | **Sample Size (n)** | **Gender (F:M, %)** | **Cancer types** | **Mean Age (Years)** | **Exercise Intervention(s)** | **Recruitment Rate (%)** |
| --- | --- | --- | --- | --- | --- | --- |
| Banck-Petersen et al (Denmark) (39) | 39 | 50 : 50 | Colorectal (100%) | 60.4 (range 37-76) | *Intervention:* Interval walking  *Control:* Waitlist control | 30 |
| Bourke et al (UK) (40) | 18 | 30 : 60 | Colorectal (100%) | 69 (range 52-80) | *Intervention:* AET & RET  *Control:* Usual care | 10.7 |
| Brown et al (USA) (41) | 39 | 62 : 38 | Colorectal (100%) | ≤70 yrs (64%)  ≥ 70 yrs (36%) | *Intervention:* AET  *Control:* Usual care | 2.8 |
| Cantarero-Villanueva et al (Spain) (42) | 46 | 35 : 65 | Colorectal (100%) | Intervention: 57 (SD 8.05)  Control: 62.31 (SD 7.93) | *Intervention:* Lumbopelvic stabilization, AET & stretching  *Control:* Usual care | 86.8 |
| Courneya et al (Canada) (43) | 102 | 42 : 58 | Colorectal (100%) | Intervention: 59.92 (SD 10.73)  Control: 61.13 (SD9.93) | *Intervention:* PA  *Control:* Waitlist control | 43 |
| Hubbard et al (UK) (44) | 41 | 34.1 : 65.9 | Colorectal (100%) | 66 (SD 11.31) | *Intervention:* Cardiac rehabilitation  *Control:* Usual care | 32.5 |

**Prostate Cancer**

| **Author (Country)** | **Sample Size (n)** | **Gender (F:M, %)** | **Cancer types** | **Mean Age (Years)** | **Exercise Intervention(s)** | **Recruitment Rate (%)** |
| --- | --- | --- | --- | --- | --- | --- |
| Campo et al (USA) (45) | 40 | 0 : 100 | Prostate (100%) | 72 (range 58-93) | *Intervention:* Qigong  *Control:* Stretching | 9.4 |
| Cormie et al (Australia) (46) | (45)20 | 0 : 100 | Prostate (100%) | Intervention: 73.1 (SD = 7.5)  Control: 71.2 (SD = 6.9) | *Intervention:* AET& RET  *Control:* Usual care | 74 |
| Courneya et al (Canada) (47) | 155 | 0 : 100 | Prostate (100%) | Intervention: 68.2 (SD 7.9)  Control: 67.7 (SD 7.5) | *Intervention:* RET  *Control:* Usual care | 30.6 |
| Kim et al (South Korea) (48) | 51 | 0 : 100 | Prostate (100%) | Intervention: 70.5 (SD 5.0)  Control: 71.0 (SD 5.5) | *Intervention:* AET & RET with an optional balance & circuit resistive calisthenics  *Control:* Stretching | 50.5 |
| Livingston et al (Australia) (49) | 147 | 0 : 100 | Prostate (100%) | 65.6 (SD 8.5) | *Intervention:* PA  *Control:* Usual care | 45.9 |
| O’Neill et al (Northern Ireland) (50) | 94 | 0 : 100 | Prostate (100%) | Intervention: 69.7 (SD 6.8)  Control: 69.9 (SD 7.0) | *Intervention:* Walking  *Control:* Usual care | 59.5 |
| Santa Mina et al (Canada) (51) | 66 | 0 : 100 | Prostate (100%) | Aerobic Group: 72.1 (8.9)  Resistance Group: 70.6 (9.5) | *Intervention:* AET *or* RET  *Control:* Waitlist control | 20.1 |
| Trinh et al (USA) (52) | 26 | 0:100 | Prostate (100%) | 65.6 (SD 6.8) | *Intervention:* AET & behavioral motivation  *Control:* AET & standard exercise therapy | 46.4 |
| Winters-Stone et al (USA) (53) | 64 | 0 :100 | Prostate (100%) | Not included | *Intervention:* RET  *Control:* Usual care | 26 |

**Endometrial Cancer**

| **Author (Country)** | **Sample Size (n)** | **Gender (F:M, %)** | **Cancer types** | **Mean Age (Years)** | **Exercise Intervention(s)** | **Recruitment Rate (%)** |
| --- | --- | --- | --- | --- | --- | --- |
| Gorzelitz et al (USA) (54) | 40 | 100:0 | Endometrial (100%) | Intervention: 60.9 (SD 9.6)  Control: 60.9 (SD 8.0) | *Intervention:* Home-based RET  *Control:* Waitlist control | 90.91 |
| Rossi et al (USA) (55) | 40 | 100 : 0 | Endometrial (100%) | Intervention: 64 (SD 10)  Control: 65 (SD 5) | *Intervention:* PA  *Control:* Waitlist control | 21.1 |

**Ovarian Cancer**

| **Author (Country)** | **Sample Size (n)** | **Gender (F:M, %)** | **Cancer types** | **Mean Age (Years)** | **Exercise Intervention(s)** | **Recruitment Rate (%)** |
| --- | --- | --- | --- | --- | --- | --- |
| Zhou et al (USA) (56) | 144 | 100 : 0 | Ovarian (100%) | 57.3 (SD 8.6) | *Intervention:* AET  *Control:* Contact control | 38.8 |

**Mixed Cancer Study Populations**

| **Author (Country)** | **Sample Size (n)** | **Gender (F:M, %)** | **Cancer types** | **Mean Age (Years)** | **Exercise Intervention(s)** | **Recruitment Rate (%)** |
| --- | --- | --- | --- | --- | --- | --- |
| Broderick et al (Ireland) (57) | 43 | 86 : 14 | Breast (72.1%), Colorectal (11.6%), Lymphoma (7%), Oesophageal (4.7%), Gynaecological (4.7%) | 51.0 (SD 9.3) | *Intervention:* AET  *Control:* Usual care | 62.3 |
| Cadmus-Bertram et al (USA) (58) | 50 | 96 : 4 | Breast (90%), Colorectal (10%) | 54.4 (SD 11.2) | *Intervention:* PA & survivorship care plan  *Control:* Survivorship care plan only | 64.1 |
| Campo et al (USA) (59) | 63 | 100 : 0 | Breast (83%), Colorectal, ovarian, cervical/uterine, thyroid, bladder, nasopharyngeal (17%) | 67 (SD 7.15) | *Intervention:* Tai Chi  *Control:* Health education | 19.5 |
| Courneya et al (Canada) (60) | 108 | 84.4 : 15.6 | Breast (40.6%), colorectal (9.4%), ovarian (5.2%), stomach (4.3%), melanoma (4.3%), Hodgkin’s lymphoma (3.1%), Brain (3.1%), Lung (3.1%), Others (15.6%), Missing (8.3%) | 51.51 (SD 10.15) | *Intervention:* AET & Group psychotherapy  *Control:* Group psychotherapy only | 80 |
| Courneya et al (UK) (61) | 122 | 41 : 59 | Indolent non-Hodgkin lymphoma (42.6%), Aggressive non-Hodgkin lymphoma (39.3%), Hodgkin lymphoma (22%) | 53.2 (range 18-80) | *Intervention:* AET  *Control:* Usual care | 17.8 |
| Crawford et al (Canada) (62) | 35 | 100 : 0 | Endometrial (40%), Ovarian (31.4%), Cervical (28.6%) | 53 (SD 11.9) | *Intervention:* Wall-climbing  *Control:* Usual care | 7.5 |
| Demark-Wahnefried et al (USA) (63) | 641 | 55.3 : 44.7 | Breast (45.3%), Prostate (39.5%) , Colorectal (15.2%) | Intervention: 73.0 (SD 5.2)  Control: 72.9 (SD 5) | *Intervention:* Exercise & diet lifestyle intervention  *Control:* Waitlist control | 3.3 |
| Donnelly et al (Northern Ireland) (64) | 33 | 100 : 0 | Ovarian (36.4%), Endometrial (33.3%), Uterine (12.1%), Cervical (12.1%), Mixed gynaecological (6.1%) | 53 (SD 10.3) | *Intervention:* Physical activity & behaviour change  *Control:* Usual care | 24.6 |
| Gothe et al (USA) (65) | 78 | 88.5:11.5 | Breast (55.55%), colorectal (3.7%) liver/bile duct (3.7%), lung (7.41%), lymphoma (7.41%), ovarian (11.11%), prostate (3.7%), skin (3.7%), thyroid (3.7%) | Intervention:  walking: 55.92 (9.2)  yoga: 55 (9.57)  Control:  stretching: 55.88 (10.7) | *Intervention:* yoga or walking  *Control:* stretching (active control) | 13.1 |
| Irwin et al (USA) (66) | 186 | 75.8 : 24.2 | Breast (52%), Lymphoma (14%), Colon (13%), Prostate (9%), Lung (4%), Endometrial (3%), Others (5%) | 59.3 (SD 10.4) | *Intervention:* AET & RET  *Control:* Waitlist control | 8 |
| Kamen et al (USA) (67) | 22 | 63.6 : 36.4 | Breast (40.9 %), Prostate (9 %), Rectal (4.5%), Testicular (4.5%), Oesophageal (4.5%), Ovarian (4.5%), Pancreatic (4.5%), Sinus (4.5%), Stomach (4.5%), Thyroid (4.5%), Tongue (4.5%) | 56 (SD 2.5) | *Intervention:* AET & RET with caregiver  *Control:* AET & RET without caregiver | 36.1 |
| Kampshoff et al (Netherlands) (68) | 277 | 80.1 : 19.9 | Breast (65.3 %), Colon (17.7 %), Lymphoma (9.4 %) , Ovarian (4.3 %), Cervix (1.4 %), Testis (1.8 %) | High-intensity group: 42 (SD 10.7)  Low-moderate intensity group: 53 (SD 11.4) | *Intervention:* AET & RET at low-moderate or high-intensity  *Control:* Waitlist control | 36.6 |
| Knobf et al (USA) (69) | 154 | 100 : 0 | Breast (83%), Gynaecological (12%), Lymphoma/other (5%) | Intervention: 50.6 (SD 5.6)  Control: 53.1 (SD 7.2) | *Intervention:* AET, RET & calcium & vitamin D supplements  *Control:* Health promotion & supplements | 33.5 |
| Knoerl et al (USA) (70) | 45 | 95:5 | Breast (32%), gynecological (41%), gastrointestinal (23%), multiple (5%) | Intervention: 60 (range 33-74)  Control: 56.5 (range 40-79.0) | *Intervention:* Yoga  *Control:* Usual care | 37.5 |
| Krull et al (USA) (71) | 70 | 100 : 0 | Leukaemia (28.4%), CNS tumour (22.4), Bone & soft tissue sarcoma (20.9%), Lymphoma (10.4%), Wilms tumour (4.5%), Other (13.4%) | Intervention: 33.0 (range 20.6 – 44.2) †  Control: 33.7 (range 21.1 – 44.9) † | *Intervention:* RET with protein supplementation  *Control:* RET & placebo | 47.6 |
| Lapen et al (USA) (72) | 42 | 100 : 0 | Breast (88.6%), Ovarian (11.4%) | Restorative yoga: 55 (range 53-60) †  Vigorous yoga: 58 (range 43-62) † | *Intervention:* Vigorous yoga  *Control:* Restorative yoga | 18.6 |
| LaStayo et al (USA)  (73) | 40 | 62.5 : 37.5 | Breast (55 %), Prostate (27.5 %), Colorectal (17.5 %), Lung (1.25 %), Lymphoma (0.25 %) | 74 (SD 6) | *Intervention:* Eccentric lower limb RET  *Control:* Usual care | 15.1 |
| Ligibel et al (USA)  (74) | 121 | 92.6 : 17.4 | Breast (82.6%), Colorectal (17.4%) | Intervention: 53.1 (SD 10.8)  Control: 55.5 (SD 10.6) | *Intervention:* PA  *Control:* Usual care | 75.2 |
| Martin et al (Australia) (75) | 159 | 45 : 55 | Prostate (54.7%), Breast (45.3%) | Prostate cancer participants: 65.8 (SD 6.6)  Breast cancer participants: 56.8 (SD 9.6) | *Intervention:* AET, RET & stretching  *Control:* Usual care | 57.9 |
| McNeely et al (Canada) (76) | 52 | 33 : 67 | Oral/oropharyngeal (62%), Larynx/hypopharynx (23%), Thyroid (4%), Other (12%) | 52 (range 32-76) | *Intervention:* Upper limb RET  *Control:* Standard therapeutic exercise | 61.9 |
| Midtgaard et al. (Denmark) (77) | 214 | 83:17 | Breast (60%), bowel (5%), ovaries (2%), uterus (2%), testes (8%), hematological (12%), other (9%) | Intervention group- 48.2  Control group- 46.2 | *Intervention:* AET and RET  *Control:* Health evaluation programme | 100 |
| O’Neill et al (Ireland) (78) | 43 | 65 : 35 | Oesophagus (27.9%), Oesophagogastric junction (62.8%), Stomach (9.3%) | Intervention: 67.19 (SD 7.49)  Control: 64.1 (SD 10.46) | *Intervention:* AET & RET  *Control:* Usual care | 40 |
| Pinto et all (USA) (79) | 20 | 95:5 | Breast (75%), other (25%) | 71.55 (SD 3.97) | *Intervention:* graduated walking programme & audiobook  *Control:* graduated walking programme only | 35.71 |
| Rastogi et al (USA) (80) | 50 | 96:4 | Breast (90%), Colorectal (10%) | 54.4 (SD 11.2) | Intervention: AET  Control: Print Material | 71.43 |
| Schwartz et al (USA) (81) | 50 | 24 : 76 | Breast (54%), Haematological (24%), Prostate (6%), Melanoma (6%), Colorectal (4%), Bladder (4%), Head & neck (2%) | 52.42 (SD 12.95) | *Intervention:* AET & RET ± Restwise online recovery programme  *Control:* No control | 75 |
| Tsai et al (USA) (82) | 25 | 86.4:13.6 | Breast (65%), Others | Clinic-Based: 56.1 (10.5)  Home-Based: 51.2 (9.5)  Control : 55.2 (13.5) | Intervention: AET  Control: Usual Care | 16.1 |
| Vallerand et al (Canada) (83) | 51 | 38 : 42 | Leukaemia (34%), Hodgkins lymphoma (23%), Non-Hodgkins lymphoma (44%) | 56.2 (SD 13.7) | *Intervention:* AET & telephone counselling  *Control:* AET only | 13.7 |
| Van De Wiel et al (Holland) (84) | 137 | 49:51 | Prostate (51%), Breast (49%) | 59.4 | Intervention: PA  Control: Usual Care | 11 |
| van Weert et al (Netherlands)  (85) | 147 | 86 : 14 | Breast (57%), Haematological (16%), Gynaecological (12%), Other (15%) | Intervention with CBT: 47.8 (SD 10.5)  Intervention without CBT: 49.9 (SD 11.3)  Control: 51.3 (SD 8.8) | *Intervention:* AET & RET ± cognitive behavioural therapy  *Control:* Waitlist control | 90.7 |
| Wurz et al (Canada) (86) | 16 | 87.5 : 12.5 | Breast (44 %), Ovarian (14.3 %), Rhabdomyosarcoma (7.1%) , Gastric (7.1%), Colorectal (7.1%), Hodgkin’s lymphoma (7.1%) , Peritoneum (7.1%), Osteosarcoma (7.1%), Soft tissue sarcoma (7.1%) | 32.84 (SD 7.93) | *Intervention:* PA  *Control:* Waitlist control | 94.1 |
| Yun et al (South Korea) (87) | 394 | 61.2 : 38.8 | Breast (35.5%), Lung (26.1%), Colorectal (21.6%), Stomach (16.8%) | With health coaching: 52.69 (SD 10.52)  Without health coaching: 54.37 (SD 11.04)  Control: 54.69 (SD 10.52) | *Intervention:* PA ± health coaching  *Control:* Usual care | 74.2 |

| AET: aerobic exercise training RET: resistance exercise training PA: physical activity †: median |
| --- |

**References**

1. Alibhai SM, O'Neill S, Fisher-Schlombs K, Breunis H, Timilshina N, Brandwein JM, et al. A pilot phase II RCT of a home-based exercise intervention for survivors of AML. Support Care Cancer. 2014;22(4):881-9.
2. Anderson RT, Kimmick GG, McCoy TP, Hopkins J, Levine E, Miller G, et al. A randomized trial of exercise on well-being and function following breast cancer surgery: the RESTORE trial. J Cancer Surviv. 2012;6(2):172-81.
3. Artese AL, Hunt RL, Ormsbee MJ, Kim JS, Arjmandi BH, Panton LB. Effect of Functional Impact Training on Body Composition, Bone Mineral Density, and Strength in Breast Cancer Survivors. Med Sci Sports Exerc. 2021;53(1):90-101.
4. Cohen J, Rogers WA, Petruzzello S, Trinh L, Mullen SP. Acute effects of aerobic exercise and relaxation training on fatigue in breast cancer survivors: A feasibility trial. Psychooncology. 2021;30(2):252-9.
5. DeMello MM, Pinto BM, Mitchell S, Dunsiger SI, Stein K. Peer support for physical activity adoption among breast cancer survivors: Do the helped resemble the helpers? Eur J Cancer Care (Engl). 2018;27(3):e12849.
6. Dieli-Conwright CM, Courneya KS, Demark-Wahnefried W, Sami N, Lee K, Buchanan TA, et al. Effects of Aerobic and Resistance Exercise on MetabolicSyndrome, Sarcopenic Obesity, and Circulating Biomarkers in Overweight or Obese Survivors of Breast Cancer: A Randomized Controlled Trial. Journal of Clinical Oncology. 2018;36.
7. dos Santos WDN, Vieira A, de Lira CAB, Mota JF, Gentil P, de Freitas Junior R, et al. Once a Week Resistance Training Improves Muscular Strength in Breast Cancer Survivors: A Randomized Controlled Trial. Integrative Cancer Therapies. 2019;18.
8. Greenlee HA, Crew KD, Mata JM, McKinley PS, Rundle AG, Zhang W, et al. A pilot randomized controlled trial of a commercial diet and exercise weight loss program in minority breast cancer survivors. Obesity (Silver Spring). 2013;21(1):65-76.
9. Irwin ML, Cadmus L, Alvarez-Reeves M, O'Neil M, Mierzejewski E, Latka R, et al. Recruiting and retaining breast cancer survivors into a randomized controlled exercise trial: the Yale Exercise and Survivorship Study. Cancer. 2008;112(11 Suppl):2593-606.
10. Irwin ML, Cartmel B, Gross CP, Ercolano E, Li F, Yao X, et al. Randomized exercise trial of aromatase inhibitor-induced arthralgia in breast cancer survivors. J Clin Oncol. 2015;33(10):1104-11.
11. Jones LM, Stoner L, Baldi JC, McLaren B. Circuit resistance training and cardiovascular health in breast cancer survivors. Eur J Cancer Care (Engl). 2020;29(4):e13231.
12. Kiecolt-Glaser JK, Bennett JM, Andridge R, Peng J, Shapiro CL, Malarkey WB, et al. Yoga's impact on inflammation, mood, and fatigue in breast cancer survivors: a randomized controlled trial. J Clin Oncol. 2014;32(10):1040-9.
13. Kim SH, Cho YU, Kim SJ, Hong S, Han MS, Choi E. The Effect on Bone Outcomes of Adding Exercise to Supplements for Osteopenic Breast Cancer Survivors: A Pilot Randomized Controlled Trial. Cancer Nurs. 2016;39(2):144-52.
14. Kim SH, Song YK, Han J, Ko YH, Lee H, Kang MJ et al. Pro-inflammatory Cytokine Levels and Cancer-related Fatigue in Breast Cancer Survivors: Effects of an Exercise Adherence Program. J Breast Cancer. 2020;23(2):205-17.
15. Littman AJ, Bertram LC, Ceballos R, Ulrich CM, Ramaprasad J, McGregor B, et al. Randomized controlled pilot trial of yoga in overweight and obese breast cancer survivors: effects on quality of life and anthropometric measures. Support Care Cancer. 2012;20(2):267-77.
16. Loh SY, Lee SY, Murray L. The Kuala Lumpur Qigong trial for women in the cancer survivorship phase-efficacy of a three-arm RCT to improve QOL. Asian Pac J Cancer Prev. 2014;15(19):8127-34.
17. Matthews CE, Wilcox S, Hanby CL, Der Ananian C, Heiney SP, Gebretsadik T, et al. Evaluation of a 12-week home-based walking intervention for breast cancer survivors. Support Care Cancer. 2007;15(2):203-11.
18. McDonald C, Bauer J, Capra S, Coll J. The muscle mass, omega-3, diet, exercise andlifestyle (MODEL) study – a randomised controlled trial for women who have completed breast cancer treatment. BMC Cancer. 2014;14.
19. Milne HM, Wallman KE, Gordon S, Courneya KS. Impact of a combined resistance and aerobic exercise program on motivational variables in breast cancer survivors: a randomized controlled trial. Ann Behav Med. 2008;36(2):158-66.
20. Owusu C, Nock NL, Feuntes V, Margevicius S, Hergenroeder P, Austin K, et al. IMPROVE, a community-based exercise intervention versus support group to improve functional and health outcomes among older African American and Non-Hispanic White breast cancer survivors from diverse socioeconomic backgrounds: Recruitment strategies and baseline characteristics. Cancer. 2021;127(11):1836-46.
21. Pinto BM, Frierson GM, Rabin C, Trunzo JJ, Marcus BH. Home-based physical activity intervention for breast cancer patients. J Clin Oncol. 2005;23(15):3577-87.
22. Pinto BM, Papandonatos GD, Goldstein MG. A randomized trial to promote physical activity among breast cancer patients. Health Psychol. 2013;32(6):616-26.
23. Rogers LQ, Courneya KS, Anton PM, Hopkins-Price P, Verhulst S, Vicari SK, et al. Effects of the BEAT Cancer physical activity behavior change intervention on physical activity, aerobic fitness, and quality of life in breast cancer survivors: a multicenter randomized controlled trial. Breast Cancer Res Treat. 2015;149(1):109-19.
24. Ruiz-Vozmediano J, Löhnchen S, Jurado L, Recio R, Rodríguez-Carrillo A, Lòpez M, et al. Influence of a Multidisciplinary Program of Diet, Exercise, and Mindfulness on the Quality of Life of Stage IIA-IIB Breast Cancer Survivors. Integrated Cancer Therapy. 2020;19.
25. Sanft T, Usiskin I, Harrigan M, Cartmel B, Lu L, Li FY, et al. Randomized controlled trial of weight loss versus usual care on telomere length in women with breast cancer: the lifestyle, exercise, and nutrition (LEAN) study. Breast Cancer Res Treat. 2018;172(1):105-12.
26. Schmitz KH, Ahmed RL, Troxel AB, Cheville A, Lewis-Grant L, Smith R, et al. Weight lifting for women at risk for breast cancer-related lymphedema: a randomized trial. JAMA. 2010;304(24):2699-705.
27. Schmitz KH, Troxel AB, Cheville A, Grant LL, Bryan CJ, Gross CR, et al. Physical Activity and Lymphedema (the PAL trial): assessing the safety of progressive strength training in breast cancer survivors. Contemp Clin Trials. 2009;30(3):233-45.
28. Sheppard VB, Hicks J, Makambi K, Hurtado-de-Mendoza A, Demark-Wahnefried W, Adams-Campbell L. The feasibility and acceptability of a diet and exercise trial in overweight and obese black breast cancer survivors: The Stepping STONE study. Contemporary clinical trials. 2016;46:106-13.
29. Stan DL, Croghan KA, Croghan IT, Jenkins SM, Sutherland SJ, Cheville AL, et al. Randomized pilot trial of yoga versus strengthening exercises in breast cancer survivors with cancer-related fatigue. Support Care Cancer. 2016;24(9):4005-15.
30. Sturgeon KM, Dean LT, Heroux M, Kane J, Bauer T, Palmer E, et al. Commercially available lifestyle modification program: randomized controlled trial addressing heart and bone health in BRCA1/2+ breast cancer survivors after risk-reducing salpingo-oophorectomy. J Cancer Surviv. 2017;11(2):246-55.
31. Taylor TR, Barrow J, Makambi K, Sheppard V, Wallington SF, Martin C, et al. A Restorative Yoga Intervention for African-American Breast Cancer Survivors: a Pilot Study. J Racial Ethn Health Disparities. 2018;5(1):62-72.
32. Thomas GA, Cartmel B, Harrigan M, Fiellin M, Capozza S, Zhou Y, et al. The effect of exercise on body composition and bone mineral density in breast cancer survivors taking aromatase inhibitors. Obesity (Silver Spring). 2017;25(2):346-51.
33. von Gruenigen V, Frasure H, Kavanagh MB, Janata J, Waggoner S, Rose P, et al. Survivors of uterine cancer empowered by exercise and healthy diet (SUCCEED): a randomized controlled trial. Gynecol Oncol. 2012;125(3):699-704.
34. Waltman NL, Twiss JJ, Ott CD, Gross GJ, Lindsey AM, Moore TE, et al. The effect of weight training on bone mineral density and bone turnover in postmenopausal breast cancer survivors with bone loss: a 24-month randomized controlled trial. Osteoporos Int. 2010;21(8):1361-9.
35. Winkels RM, Sturgeon KM, Kallan MJ, Dean LT, Zhang Z, Evangelisti M, et al. The women in steady exercise research (WISER) survivor trial: The innovative transdisciplinary design of a randomized controlled trial of exercise and weight-loss interventions among breast cancer survivors with lymphedema. Contemp Clin Trials. 2017;61:63-72.
36. Sturgeon KM, Hackley R, Fornash A, Dean LT, Laudermilk M, Brown JC, et al. Strategic recruitment of an ethnically diverse cohort of overweight survivors of breast cancer with lymphedema. Cancer. 2018;124(1):95-104.
37. Winters-Stone KM, Dobek J, Nail LM, Bennett JA, Leo MC, Torgrimson-Ojerio B, et al. Impact + resistance training improves bone health and body composition in prematurely menopausal breast cancer survivors: a randomized controlled trial. Osteoporos Int. 2013;24(5):1637-46.
38. Winters-Stone KM, Dobek J, Bennett JA, Nail LM, Leo MC, Schwartz A. The effect of resistance training on muscle strength and physical function in older, postmenopausal breast cancer survivors: a randomized controlled trial. J Cancer Surviv. 2012;6(2):189-99.
39. Banck-Petersen A, Olsen CK, Djurhuus SS, Herrstedt A, Thorsen-Streit S, Ried-Larsen M, et al. The "Interval Walking in Colorectal Cancer" (I-WALK-CRC) study: Design, methods and recruitment results of a randomized controlled feasibility trial. Contemp Clin Trials Commun. 2018;9:143-50.
40. Bourke L, Thompson G, Gibson DJ, Daley A, Crank H, Adam I, et al. Pragmatic lifestyle intervention in patients recovering from colon cancer: a randomized controlled pilot study. Arch Phys Med Rehabil. 2011;92(5):749-55.
41. Brown JC, Troxel AB, Ky B, Damjanov N, Zemel BS, Rickels MR, et al. A randomized phase II dose-response exercise trial among colon cancer survivors: Purpose, study design, methods, and recruitment results. Contemporary clinical trials. 2016;47:366-75.
42. Cantarero-Villanueva I, Sanchez-Jimenez A, Galiano-Castillo N, Diaz-Rodriguez L, Martin-Martin L, Arroyo-Morales M. Effectiveness of Lumbopelvic Exercise in Colon Cancer Survivors: A Randomized Controlled Clinical Trial. Med Sci Sports Exerc. 2016;48(8):1438-46.
43. Courneya KS, Friedenreich CM, Quinney HA, Fields ALA, Jones LW, Fairey AS. A randomized trial of exercise and quality of life in colorectal cancer survivors. European Journal of Cancer. 2003;12:347-57.
44. Hubbard G, Munro J, O'Carroll R, Mutrie N, Kidd L, Haw S, et al. The use of cardiac rehabilitation services to aid the recovery of patients with bowel cancer: a pilot randomised controlled trial with embedded feasibility study. Health Services and Delivery Research. Southampton (UK)2016.
45. Campo RA, Agarwal N, LaStayo PC, O'Connor K, Pappas L, Boucher KM, et al. Levels of fatigue and distress in senior prostate cancer survivors enrolled in a 12-week randomized controlled trial of Qigong. J Cancer Surviv. 2014;8(1):60-9.
46. Cormie P, Newton RU, Spry N, Joseph D, Taaffe DR, Galvao DA. Safety and efficacy of resistance exercise in prostate cancer patients with bone metastases. Prostate Cancer Prostatic Dis. 2013;16(4):328-35.
47. Courneya KS, Segal RJ, Reid RD, Jones LW, Malone SC, Venner PM, et al. Three independent factors predicted adherence in a randomized controlled trial of resistance exercise training among prostate cancer survivors. J Clin Epidemiol. 2004;57(6):571-9.
48. Kim SH, Seong DH, Yoon SM, Choi YD, Choi E, Song Y, et al. The Effect on Bone Outcomes of Home-based Exercise Intervention for Prostate Cancer Survivors Receiving Androgen Deprivation Therapy: A Pilot Randomized Controlled Trial. Cancer Nurs. 2018;41(5):379-88.
49. Livingston PM, Craike MJ, Salmon J, Courneya KS, Gaskin CJ, Fraser SF, et al. Effects of a clinician referral and exercise program for men who have completed active treatment for prostate cancer: A multicenter cluster randomized controlled trial (ENGAGE). Cancer. 2015;121(15):2646-54.
50. O'Neill RF, Haseen F, Murray LJ, O'Sullivan JM, Cantwell MM. A randomised controlled trial to evaluate the efficacy of a 6-month dietary and physical activity intervention for patients receiving androgen deprivation therapy for prostate cancer. J Cancer Surviv. 2015;9(3):431-40.
51. Santa Mina D, Alibhai SMH, Matthew AG, Guglietti CL, Pirbaglou M, Trachtenberg J, et al. A Randomized Trial of Aerobic Versus Resistance Exercise in Prostate Cancer Survivors. Journal of Aging and Physical Activity. 2013;21.
52. Trinh L, Kramer AF, Rowland K, Strom DA, Wong JN, McAuley E. A pilot feasibility randomized controlled trial adding behavioral counseling to supervised physical activity in prostate cancer survivors: behavior change in prostate cancer survivors trial (BOOST). J Behav Med. 2021;44(2):172-86.
53. Winters-Stone KM, Lyons KS, Nail LM, Beer TM. The Exercising Together project: design and recruitment for a randomized, controlled trial to determine the benefits of partnered strength training for couples coping with prostate cancer. Contemp Clin Trials. 2012;33(2):342-50.
54. Gorzelitz J, Costanzo E, Gangnon R, Koltyn K, Dietz AT, Spencer RJ, et al. Feasibility and acceptability of home-based strength training in endometrial cancer survivors. J Cancer Surviv. 2021.
55. Rossi A, Garber CE, Ortiz M, Shankar V, Goldberg GL, Nevadunsky NS. Feasibility of a physical activity intervention for obese, socioculturally diverse endometrial cancer survivors. Gynecol Oncol. 2016;142(2):304-10.
56. Zhou Y, Cartmel B, Gottlieb L, Ercolano EA, Li F, Harrigan M, et al. Randomized Trial of Exercise on Quality of Life in Women With Ovarian Cancer: Women's Activity and Lifestyle Study in Connecticut (WALC). J Natl Cancer Inst. 2017;109(12).
57. Broderick JM, Guinan E, Kennedy MJ, Hollywood D, Courneya KS, Culos-Reed SN, et al. Feasibility and efficacy of a supervised exercise intervention in de-conditioned cancer survivors during the early survivorship phase: the PEACH trial. J Cancer Surviv. 2013;7(4):551-62.
58. Cadmus-Bertram L, Tevaarwerk AJ, Sesto ME, Gangnon R, Van Remortel B, Date P. Building a physical activity intervention into clinical care for breast and colorectal cancer survivors in Wisconsin: a randomized controlled pilot trial. J Cancer Surviv. 2019;13(4):593-602.
59. Campo RA, O'Connor K, Light KC, Nakamura Y, Lipschitz DL, LaStayo PC, et al. Feasibility and acceptability of a Tai Chi Chih randomized controlled trial in senior female cancer survivors. Integr Cancer Ther. 2013;12(6):464-74.
60. Courneya KS, Friedenreich CM, Sela RA, Quinney HA, Rhodes RE, Handman M. The group psychotherapy and home-based physical exercise (group-hope) trial in cancer survivors: physical fitness and quality of life outcomes. Psychooncology. 2003;12(4):357-74.
61. Courneya KS, Sellar CM, Stevinson C, McNeely ML, Peddle CJ, Friedenreich CM, et al. Randomized controlled trial of the effects of aerobic exercise on physical functioning and quality of life in lymphoma patients. J Clin Oncol. 2009;27(27):4605-12.
62. Crawford JJ, Vallance JK, Holt NL, Bell GJ, Steed H, Courneya KS. A Pilot Randomized, Controlled Trial of a Wall Climbing Intervention for Gynecologic Cancer Survivors. Oncol Nurs Forum. 2017;44(1):77-86.
63. Demark-Wahnefried W, Morey MC, Sloane R, Snyder DC, Miller PE, Hartman TJ, et al. Reach out to enhance wellness home-based diet-exercise intervention promotes reproducible and sustainable long-term improvements in health behaviors, body weight, and physical functioning in older, overweight/obese cancer survivors. J Clin Oncol. 2012;30(19):2354-61.
64. Donnelly CM, Blaney JM, Lowe-Strong A, Rankin JP, Campbell A, McCrum-Gardner E, et al. A randomised controlled trial testing the feasibility and efficacy of a physical activity behavioural change intervention in managing fatigue with gynaecological cancer survivors. Gynecol Oncol. 2011;122(3):618-24.
65. Gothe NP, Erlenbach E. Feasibility of a yoga, aerobic and stretching-toning exercise program for adult cancer survivors: the STAYFit trial. J Cancer Surviv. 2021.
66. Irwin ML, Cartmel B, Harrigan M, Li F, Sanft T, Shockro L, et al. Effect of the LIVESTRONG at the YMCA exercise program on physical activity, fitness, quality of life, and fatigue in cancer survivors. Cancer. 2017;123(7):1249-58.
67. Kamen C, Heckler C, Janelsins MC, Peppone LJ, McMahon JM, Morrow GR, et al. A Dyadic Exercise Intervention to Reduce Psychological Distress Among Lesbian, Gay, and Heterosexual Cancer Survivors. LGBT Health. 2016;3(1):57-64.
68. Kampshoff CS, Chinapaw MJ, Brug J, Twisk JW, Schep G, Nijziel MR, et al. Randomized controlled trial of the effects of high intensity and low-to-moderate intensity exercise on physical fitness and fatigue in cancer survivors: results of the Resistance and Endurance exercise After ChemoTherapy (REACT) study. BMC Med. 2015;13:275.
69. Knobf MT, Jeon S, Smith B, Harris L, Kerstetter J, Thompson AS, et al. Effect of a randomized controlled exercise trial on bone outcomes: influence of adjuvant endocrine therapy. Breast Cancer Res Treat. 2016;155(3):491-500.
70. Knoerl R, Giobbie-Hurder A, Berfield J, Berry D, Meyerhardt JA, Wright AA, et al. Yoga for chronic chemotherapy-induced peripheral neuropathy pain: a pilot, randomized controlled trial. J Cancer Surviv. 2022;16(4):882-91.
71. Krull MR, Howell CR, Partin RE, Lanctot J, Phipps S, Klosky JL, et al. Protein Supplementation and Resistance Training in Childhood Cancer Survivors. Med Sci Sports Exerc. 2020;52(10):2069-77.
72. Lapen K, Benusis L, Pearson S, Search B, Coleton M, Li QS, et al. A Feasibility Study of Restorative Yoga Versus Vigorous Yoga Intervention for Sedentary Breast and Ovarian Cancer Survivors. Int J Yoga Therap. 2018;28(1):79-85.
73. LaStayo PC, Marcus RL, Dibble LE, Smith SB, Beck SL. Eccentric exercise versus usual-care with older cancer survivors: the impact on muscle and mobility--an exploratory pilot study. BMC Geriatr. 2011;11:5.
74. Ligibel JA, Meyerhardt J, Pierce JP, Najita J, Shockro L, Campbell N, et al. Impact of a telephone-based physical activity intervention upon exercise behaviors and fitness in cancer survivors enrolled in a cooperative group setting. Breast Cancer Res Treat. 2012;132(1):205-13.
75. Martin EA, Battaglini CL, Hands B, Naumann F. Higher-Intensity Exercise Results in More Sustainable Improvements for VO2peak for Breast and Prostate Cancer Survivors. Oncology nursing forum. 2015;42(3):241-9.
76. McNeely ML, Parliament MB, Seikaly H, Jha N, Magee DJ, Haykowsky MJ, et al. Effect of exercise on upper extremity pain and dysfunction in head and neck cancer survivors: a randomized controlled trial. Cancer. 2008;113(1):214-22.
77. Midtgaard J, Christensen JF, Tolver A, Jones LW, Uth J, Rasmussen B, et al. Efficacy of multimodal exercise-based rehabilitation on physical activity, cardiorespiratory fitness, and patient-reported outcomes in cancer survivors: a randomized, controlled trial. Ann Oncol. 2013;24(9):2267-73.
78. O'Neill LM, Guinan E, Doyle SL, Bennett AE, Murphy C, Elliott JA, et al. The RESTORE Randomized Controlled Trial: Impact of a Multidisciplinary Rehabilitative Program on Cardiorespiratory Fitness in Esophagogastric cancer Survivorship. Annals of surgery. 2018;268(5):747-55.
79. Pinto BM, Kindred M, Franco R, Simmons V, Hardin J. A 'novel' multi-component approach to promote physical activity among older cancer survivors: a pilot randomized controlled trial. Acta Oncol. 2021;60(8):968-75.
80. Rastogi S, Tevaarwerk AJ, Sesto M, Van Remortel B, Date P, Gangnon R, et al. Effect of a technology-supported physical activity intervention on health-related quality of life, sleep, and processes of behavior change in cancer survivors: A randomized controlled trial. Psychooncology. 2020;29(11):1917-26.
81. Schwartz AL, Biddle-Newberry M, de Heer HD. Randomized trial of exercise and an online recovery tool to improve rehabilitation outcomes of cancer survivors. Phys Sportsmed. 2015;43(2):143-9.
82. Tsai E, Mouhayar E, Lenihan D, Song J, Durand JB, Fadol A, et al. Feasibility and Outcomes of an Exercise Intervention for Chemotherapy-Induced Heart Failure. J Cardiopulm Rehabil Prev. 2019;39(3):199-203.
83. Vallerand JR, Rhodes RE, Walker GJ, Courneya KS. Feasibility and preliminary efficacy of an exercise telephone counseling intervention for hematologic cancer survivors: a phase II randomized controlled trial. J Cancer Surviv. 2018;12(3):357-70.
84. van de Wiel HJ, Stuiver MM, May AM, van Grinsven S, Aaronson NK, Oldenburg HSA, et al. Effects of and Lessons Learned from an Internet-Based Physical Activity Support Program (with and without Physiotherapist Telephone Counselling) on Physical Activity Levels of Breast and Prostate Cancer Survivors: The PABLO Randomized Controlled Trial. Cancers (Basel). 2021;13(15).
85. van Weert E, May AM, Korstjens I, Post WJ, van der Schans CP, van den Borne B, et al. Cancer-related fatigue and rehabilitation: a randomized controlled multicenter trial comparing physical training combined with cognitive-behavioral therapy with physical training only and with no intervention. Phys Ther. 2010;90(10):1413-25.
86. Wurz A, Brunet J. Exploring the feasibility and acceptability of a mixed-methods pilot randomized controlled trial testing a 12-week physical activity intervention with adolescent and young adult cancer survivors. Pilot Feasibility Stud. 2019;5:154.
87. Yun YH, Lim CI, Lee ES, Kim YT, Shin KH, Kim YW, et al. Efficacy of health coaching and a web-based program on physical activity, weight, and distress management among cancer survivors: A multi-centered randomised controlled trial. Psychooncology. 2020;29(7):1105-14.
