# Supplementary material for: Recruitment rates and strategies in exercise trials in cancer survivorship: a systematic review
Source: J Cancer Surviv. 2023 Apr 6;18(4):1233–42. doi: 10.1007/s11764-023-01363-8 (PMC11324688; doi:10.1007/s11764-023-01363-8)
Supplement: Supplementary file 3 — Supplementary file3 (DOCX 44 KB) [file 11764_2023_1363_MOESM3_ESM.docx]

**Supplemental Material 3 – List of Excluded Reports and Reasons for Exclusion**

**Title:** Recruitment Rates and Strategies in Exercise Trials in Cancer Survivorship - A Systematic Review

**Authors:** Sophie Reynolds^1,2^, Louise O’Connor^2,3^, Anna McGee^1,2^, Anna Quinn Kilcoyne^1,2^, Archie Connolly^1,2^, David Mockler^4^, Emer Guinan^1,2^, Linda O’Neill^2,3^

**Affiliations:**

^1^ School of Medicine, Trinity College Dublin, the University of Dublin, Dublin, Ireland

^2^ Trinity St James’s Cancer Institute, Dublin, Ireland

^3^ Discipline of Physiotherapy, School of Medicine, Trinity College Dublin, the University of Dublin, Dublin, Ireland

^4^ John Stearne Library, Trinity Centre for Health Sciences, St James’s Hospital, Dublin, Ireland

**Corresponding Author:**

Dr Linda O’Neill

Discipline of Physiotherapy,

Trinity Centre for Health Sciences, St James’s Hospital,

Dublin 8

Email: [oneilll8@tcd.ie](mailto:oneilll8@tcd.ie)

Telephone: +353 1 8964809

**Supplemental Material 3** **– List of Excluded Reports and Reasons for Exclusion**

|  | **Reference** | **Reason for Exclusion** |
| --- | --- | --- |
| 1 | Adams BD, Arem H, Hubal MJ, Cartmel B, Li F, Harrigan M, Sanft T, Cheng CJ, Pusztai L, Irwin ML. Exercise and weight loss interventions and miRNA expression in women with breast cancer. Breast Cancer Res Treat. 2018 Jul;170(1):55-67. doi: 10.1007/s10549-018-4738-6. Epub 2018 Mar 6. PMID: 29511965; PMCID: PMC6444907. | Wrong intervention |
| 2 | Ahmed RL, Thomas W, Yee D, Schmitz KH. Randomized controlled trial of weight training and lymphedema in breast cancer survivors. J Clin Oncol. 2006 Jun 20;24(18):2765-72. doi: 10.1200/JCO.2005.03.6749. Epub 2006 May 15. Erratum in: J Clin Oncol. 2006 Aug 1;24(22):3716. PMID: 16702582. | Secondary paper |
| 3 | Aycinena AC, Valdovinos C, Crew KD, Tsai WY, Mata JM, Sandoval R, Hershman D, Greenlee H. Barriers to Recruitment and Adherence in a Randomized Controlled Diet and Exercise Weight Loss Intervention Among Minority Breast Cancer Survivors. J Immigr Minor Health. 2017 Feb;19(1):120-129. doi: 10.1007/s10903-015-0310-1. PMID: 26801931. | Secondary paper |
| 4 | Bade BC, Gan G, Li F et al. Randomized trial of physical activity on quality of life and lung cancer biomarkers in patients with advanced stage lung cancer: a pilot study. BMC Cancer. 2021; 21, 352. https://doi.org/10.1186/s12885-021-08084-0 | Wrong patient population |
| 5 | Baglia ML, Lin IH, Cartmel B, Sanft T, Ligibel J, Hershman DL, Harrigan M, Ferrucci LM, Li FY, Irwin ML, Endocrine-related quality of life in a randomized trial of exercise on aromatase inhibitor–induced arthralgias in breast cancer survivors. Cancer. 2019; 125: 2262-2271. https://doi.org/10.1002/cncr.32051 | Wrong intervention |
| 6 | O'Carroll Bantum E, Albright C, White K, Berenberg J, Layi G, Ritter P, Laurent D, Plant K, Lorig K. Surviving and Thriving With Cancer Using a Web-Based Health Behavior Change Intervention: Randomized Controlled Trial  J Med Internet Res. 2014;16(2):e54. DOI: 10.2196/jmir.3020 | Wrong intervention |
| 7 | Bao T, Zhi I, Baser R, Hooper M, Chen C, Piulson L, Li QS, Galantino ML, Blinder V, Robson M, Seidman A, Panageas KS, Mao JJ. Yoga for Chemotherapy-Induced Peripheral Neuropathy and Fall Risk: A Randomized Controlled Trial. JNCI Cancer Spectr. 2020 Jun 4;4(6):pkaa048. doi: 10.1093/jncics/pkaa048. PMID: 33225208; PMCID: PMC7666827. | Insufficient recruitment details |
| 8 | Cadmus Bertram LA, Chung G, Yu H, Salovey P, Irwin M. Feasibility of institutional registry-based recruitment for enrolling newly diagnosed breast cancer patients in an exercise trial. J Phys Act Health. 2011 Sep;8(7):955-63. doi: 10.1123/jpah.8.7.955. PMID: 21885886. | Primary treatment not completed |
| 9 | Blair C, Harding E, Wiggins C, Kang H, Schwartz M, Tarnower A, Du R, Kinney A. A Home-Based Mobile Health Intervention to Replace Sedentary Time With Light Physical Activity in Older Cancer Survivors: Randomized Controlled Pilot Trial. JMIR Cancer. 2021;7(2):e18819. DOI: 10.2196/18819. | Wrong intervention |
| 10 | Booth CM, Vardy JL, O'Callaghan CJ, Gill S, Jonker DJ, Friedenreich C, et al. A phase III study of the impact of a physical activity program on disease-free survival in patients with high-risk stage II or stage III colon cancer: A randomized controlled trial (NCIC CTG CO.21). Journal of Clinical Oncology. 2015; 33(15_suppl):TPS3620-TPS. | Abstract only |
| 11 | Brown JC, Damjanov N, Courneya KS, Troxel AB, Zemel BS, Rickels MR, Ky B, Rhim AD, Rustgi AK, Schmitz KH. A randomized dose-response trial of aerobic exercise and health-related quality of life in colon cancer survivors. Psycho-oncology. 2018Apr;27(4):1221-1228. doi: 10.1002/pon.4655. Epub 2018 Feb 20. PMID: 29388275; PMCID: PMC5895514. | Secondary paper |
| 12 | Bucciarelli V, Bianco F, Di Blasio A, Morano T, Tuosto D, Mucedola F, Izzicup P, Napolitano G, Bucci I, Di Baldassarre A, et al. The effects of adherence to physical exercise on cardiovascular efficiency in breast cancer survivors. Giornale italiano di cardiologia. 2019; 20 (12), CN-02094973 | Abstract only |
| 13 | Cadmus LA, Salovey P, Yu H, Chung G, Kasl S, Irwin, ML. Exercise and quality of life during and after treatment for breast cancer: results of two randomized controlled trials. Psycho-Oncology. 2009; 18: 343-352. https://doi.org/10.1002/pon.1525 | Secondary paper |
| 14 | Cadmus-Bertram L, Littman AJ, Ulrich CM, Stovall R, Ceballos RM, McGregor BA, et al. Predictors of Adherence to a 26-Week Viniyoga Intervention Among Post-Treatment Breast Cancer Survivors. The Journal of Alternative and Complementary Medicine. 2013;19(9):751-8. | Wrong study design |
| 15 | Cantarero-Villanueva I, Cuesta-Vargas AI, Lozano-Lozano M, Fernández-Lao C, Fernández-Pérez A, Galiano-Castillo N. Changes in Pain and Muscle Architecture in Colon Cancer Survivors After a Lumbopelvic Exercise Program: A Secondary Analysis of a Randomized Controlled Trial. Pain Medicine. 2017;18(7):1366-76. | Secondary paper |
| 16 | Cartmel B, Hughes M, Ercolano EA, Gottlieb L, Li F, Zhou Y, Harrigan M, Ligibel JA, von Gruenigen VE, Gogoi R, Schwartz PE, Risch HA, Lu L, Irwin ML. Randomized trial of exercise on depressive symptomatology and brain derived neurotrophic factor (BDNF) in ovarian cancer survivors: The Women’s Activity and Lifestyle Study in Connecticut (WALC). Gynecol Oncol. 2021 May;161(2):587-594. doi: 10.1016/j.ygyno.2021.02.036. Epub 2021 Mar 26. PMID: 33773809; PMCID: PMC8085084. | Secondary paper |
| 17 | Cheville AL, Kollasch J, Vandenberg J, Shen T, Grothey A, Gamble G, Basford JR. A home-based exercise program to improve function, fatigue, and sleep quality in patients with Stage IV lung and colorectal cancer: a randomized controlled trial. J Pain Symptom Manage. 2013 May;45(5):811-21. doi: 10.1016/j.jpainsymman.2012.05.006. Epub 2012 Sep 24. PMID: 23017624; PMCID: PMC4524515. | Wrong patient population |
| 18 | Cormie P, Newton RU, Spry N, Joseph D, Taaffe DR, Galvão DA. Safety and efficacy of resistance exercise in prostate cancer patients with bone metastases. Prostate Cancer Prostatic Dis. 2013 Dec;16(4):328-35. doi: 10.1038/pcan.2013.22. Epub 2013 Aug 6. Erratum in: Prostate Cancer Prostatic Dis. 2015 Jun;18(2):196. PMID: 23917308. | Wrong patient population |
| 19 | Courneya KS, Friedenreich CM, Quinney HA, Fields ALA, Jones LW, Fairey AS. Predictors of adherence and contamination in a randomized trial of exercise in colorectal cancer survivors. Psycho-Oncology, 2004; 13: 857-866. https://doi.org/10.1002/pon.802 | Secondary paper |
| 20 | Courneya KS, Friedenreich CM, Sela RA, Quinney HA, Rhodes RE, Jones LW. Exercise motivation and adherence in cancer survivors after participation in a randomized controlled trial: an attribution theory perspective. Int J Behav Med. 2004;11(1):8-17. doi: 10.1207/s15327558ijbm1101_2. PMID: 15194515. | Secondary paper |
| 21 | Courneya KS, Segal RJ, Reid RD, Jones LW, Malone SC, Venner PM, Parliament MB, Scott CG, Quinney HA, Wells GA. Three independent factors predicted adherence in a randomized controlled trial of resistance exercise training among prostate cancer survivors. J Clin Epidemiol. 2004 Jun; 57(6):571-9. doi: 10.1016/j.jclinepi.2003.11.010. PMID: 15246125. | Secondary paper |
| 22 | Courneya KS, Vardy JL, O'Callaghan CJ, Friedenreich CM, Campbell KL, Prapavessis H, Crawford JJ, O'Brien P, Dhillon HM, Jonker DJ, Chua NS, Lupichuk S, Sanatani MS, Gill S, Meyer RM, Begbie S, Bonaventura T, Burge ME, Turner J, Tu D, Booth CM. Effects of a Structured Exercise Program on Physical Activity and Fitness in Colon Cancer Survivors: One Year Feasibility Results from the CHALLENGE Trial. Cancer Epidemiol Biomarkers Prev. 2016 Jun; 25(6):969-77. doi: 10.1158/1055-9965.EPI-15-1267. Epub 2016 Apr 8. PMID: 27197271. | Secondary paper |
| 23 | Courneya, K.S., Vardy, J., Gill, S., Jonker, D., O'Brien, P., Friedenreich, C.M., Dhillon, H., Wong, R.K.S., Meyer, R.M., Crawford, J.J., Campbell, K.L., Prapavessis, H., O'Callaghan, C., Turner, J., Spencer, L.M., van der Ploeg, H.P., Tu, D., & Booth, C.M. Update on the colon health and life-long exercise change (CHALLENGE) trial: A phase III study of the impact of an exercise program on disease-free survival in colon cancer survivors. Current Colorectal Cancer Reports. 2014;10(3). | Secondary paper |
| 24 | Courneya KS, Friedenreich CM, Sela RA, Quinney HA, Rhodes RE. Correlates of adherence and contamination in a randomized controlled trial of exercise in cancer survivors: an application of the theory of planned behavior and the five factor model of personality. Ann Behav Med. 2002 Fall; 24(4):257-68. doi: 10.1207/S15324796ABM2404_02. PMID: 12434937. | Secondary paper |
| 25 | Courneya KS, Stevinson C, McNeely M, Sellar C, Friedenreich C, Peddle-McIntyre C, Chua N, Reiman T. Effects of Supervised Exercise on Motivational Outcomes and Longer-Term Behavior. Medicine & Science in Sports & Exercise. March 2012; 44(3):p 542-549. DOI: 10.1249/MSS.0b013e3182301e06.. | Secondary paper |
| 26 | Cuesta-Vargas AI, Buchan J, Arroyo-Morales M. Plus deep water running in cancer-related fatigue. Eur J Cancer Care (Engl). 2014; 23: 15-21. https://doi.org/10.1111/ecc.12114. | Wrong study design |
| 27 | Danhauer SC, Mihalko SL, Russell GB, Campbell CR, Felder L, Daley K, Levine EA. Restorative yoga for women with breast cancer: findings from a randomized pilot study. Psycho-Oncology. 2009;18: 360-368. https://doi.org/10.1002/pon.1503. | Primary treatment not completed |
| 28 | Demark-Wahnefried W, Morey MC, Clipp EC, Pieper CF, Snyder DC, Sloane R, et al. Leading the Way in Exercise and Diet (Project LEAD): intervening to improve function among older breast and prostate cancer survivors. Controlled Clinical Trials. 2003; 24(2):206-23. | Wrong intervention |
| 29 | Derry HM, Jaremka LM, Bennett JM, Peng J, Andridge R, Shapiro C, Malarkey WB, Emery CF, Layman R, Mrozek E, Glaser R, Kiecolt-Glaser JK.Yoga and self-reported cognitive problems in breast cancer survivors: a randomized controlled trial. Psycho-Oncology. 2015; 24: 958– 966. doi: 10.1002/pon.3707. | Secondary paper |
| 30 | Di Blasio A, Morano T, Napolitano G, Bucci I, Di Santo S, Gallina S, et al. Nordic Walking and the Isa Method for Breast Cancer Survivors: Effects on Upper Limb Circumferences and Total Body Extracellular Water - a Pilot Study. Breast Care. 2016;11(6):428-31. | Insufficient recruitment details |
| 31 | Dieli-Conwright CM, Hughes-Parmentier J, Lee K, Spicer D, Mack W, Sattler F, Mittelman SD. Abstract 985: Adipose tissue inflammation in breast cancer survivors: Effects of a 16-week aerobic and resistance exercise intervention. Cancer Res. 2017; 77 (13_Supplement): 985. https://doi.org/10.1158/1538-7445.AM2017-985. | Abstract only |
| 32 | Donnelly C, Blaney J, Lowe A, Rankin J, Campbell A, Gracey JA feasibility trial of a home based walking intervention in managing fatigue with gynaecological cancer survivors. Supportive Care in Cancer. 2011; 19(2), S263, https://doi. 10.1007/s00520-011-1184-y | Abstract only |
| 33 | Eisele M, Twomey R, Pohl AJ, et al. The online delivery of exercise oncology classes supported with health coaching: A pilot randomized controlled trial. medRxiv; 2021; DOI: 10.1101/2021.11.29.21266169. | Insufficient recruitment details |
| 34 | Fernández-Lao C, Cantarero-Villanueva I, Ariza-Garcia A, Courtney C, Fernández-de-las-Peñas C, Arroyo-Morales M. Water versus land-based multimodal exercise program effects on body composition in breast cancer survivors: a controlled clinical trial. Support Care Cancer. 2013 Feb;21(2):521-30. doi: 10.1007/s00520-012-1549-x. Epub 2012 Aug 4. PMID: 22864470. | Wrong study design |
| 35 | Ferrucci LM, Cartmel B, Harrigan M, Sanft T, Playdon M, Jia W, Yu H, Johnson CH, Pusztai L, Chagpar AB, Irwin ML. Abstract 5321: Metabolomics and body mass index among breast cancer survivors in The Lifestyle, Exercise, and Nutrition (LEAN) Study. Cancer Res 1 July 2017; 77 (13_Supplement): 5321. https://doi.org/10.1158/1538-7445.AM2017-5321. | Abstract only |
| 36 | Forbes C, Blanchard C, Mummery W, Courneya K. Feasibility and Preliminary Efficacy of an Online Intervention to Increase Physical Activity in Nova Scotian Cancer Survivors: A Randomized Controlled Trial. JMIR Cancer. 2015;1(2):e12. DOI: 10.2196/cancer.4586. | Wrong intervention |
| 37 | Golsteijn R, Bolman C, Peels D, Volders E, de Vries H, Lechner L. A Web-Based and Print-Based Computer-Tailored Physical Activity Intervention for Prostate and Colorectal Cancer Survivors: A Comparison of User Characteristics and Intervention Use. J Med Internet Res. 2017;19(8):e298. DOI: 10.2196/jmir.7838 | Wrong patient population |
| 38 | Gothe NP, Erlenbach ED, Streeter SL, et al. Effects of yoga, aerobic, and stretching and toning exercises on cognition in adult cancer survivors: protocol of the STAY Fit pilot randomized controlled trial. Trials. 2020; 21, 792. https://doi.org/10.1186/s13063-020-04723-2. | Protocol paper only |
| 39 | Guest DD, Evans EM and Rogers LQ. Diet components associated with perceived fatigue in breast cancer survivors. European Journal of Cancer Care. 2013; 22: 51-59. https://doi.org/10.1111/j.1365-2354.2012.01368.x. | Wrong intervention |
| 40 | Guinan E, Hussey J, Broderick JM, Lithander FE, O'Donnell D, Kennedy MJ, Connolly EM. The effect of aerobic exercise on metabolic and inflammatory markers in breast cancer survivors--a pilot study. Support Care Cancer. 2013 Jul;21(7):1983-92. doi: 10.1007/s00520-013-1743-5. Epub 2013 Feb 22. PMID: 23430010. | Secondary paper |
| 41 | Hadrabova M, Janikova A, Hrncirikova I, Svobodová Z, Mala A, Kapounková K, et al. Sympathovagal imbalance in lymphoma patients can be restored by physical training – single centre prospective trial. Hematological Oncology. 2019; 37:546-7. | Abstract only |
| 42 | Hardcastle SJ, Jiménez-Castuera R, Maxwell-Smith C, Bulsara MK, Hince D. Fitbit wear-time and patterns of activity in cancer survivors throughout a physical activity intervention and follow-up: Exploratory analysis from a randomised controlled trial. PLoS One. 2020 Oct 19;15(10):e0240967. doi: 10.1371/journal.pone.0240967. PMID: 33075100; PMCID: PMC7571692. | Secondary paper |
| 43 | Hirschey, R, Kimmick, G, Hockenberry, M, et al. A randomized phase II trial of MOVING ON: An intervention to increase exercise outcome expectations among breast cancer survivors. Psycho-Oncology. 2018; 27: 2450– 2457. https://doi.org/10.1002/pon.4849 | Wrong intervention |
| 44 | Hubbard G, Adams R, Campbell A, Kidd L, Leslie SJ, Munro J, Watson A. Is referral of postsurgical colorectal cancer survivors to cardiac rehabilitation feasible and acceptable? A pragmatic pilot randomised controlled trial with embedded qualitative study. BMJ Open. 2016 Jan 4;6(1):e009284. doi: 10.1136/bmjopen-2015-009284. PMID: 26729381; PMCID: PMC4716214. | Primary treatment not completed |
| 45 | Irwin ML, Cadmus L, Alvarez-Reeves M, O'Neil M, Mierzejewski E, Latka R, Yu H, Dipietro L, Jones B, Knobf MT, Chung GG, Mayne ST. Recruiting and retaining breast cancer survivors into a randomized controlled exercise trial: the Yale Exercise and Survivorship Study. Cancer. 2008 Jun 1;112(11 Suppl):2593-606. doi: 10.1002/cncr.23446. Erratum in: Cancer. 2008 Oct 1;113(7):1716. PMID: 18428192; PMCID: PMC5450159. | Duplicate |
| 46 | Irwin ML, Cadmus L, Alvarez-Reeves M, O'Neil M, Mierzejewski E, Latka R, Yu H, Dipietro L, Jones B, Knobf MT, Chung GG, Mayne ST. Recruiting and retaining breast cancer survivors into a randomized controlled exercise trial: the Yale Exercise and Survivorship Study. Cancer. 2008 Jun 1;112(11 Suppl):2593-606. doi: 10.1002/cncr.23446. Erratum in: Cancer. 2008 Oct 1;113(7):1716. PMID: 18428192; PMCID: PMC5450159. | Duplicate |
| 47 | Irwin, ML, Alvarez-Reeves M, Cadmus L, Mierzejewski E, Mayne ST, Yu H, Chung, GG, Jones B, Knobf MT, DiPietro L. Exercise Improves Body Fat, Lean Mass, and Bone Mass in Breast Cancer Survivors. Obesity. 2009; 17: 1534-1541. https://doi.org/10.1038/oby.2009.18 | Secondary paper |
| 48 | Iyer NS, Cartmel B, Friedman L, Li F, Zhou Y, Ercolano E, Harrigan M, Gottlieb L, McCorkle R, Schwartz PE, Irwin ML . Lymphedema in ovarian cancer survivors: Assessing diagnostic methods and the effects of physical activity. Cancer. (2018)124: 1929-1937. https://doi.org/10.1002/cncr.31239 | Secondary paper |
| 49 | James E, Boyes A, Courbeya K, Lubans D, Stacey F, Morgan P, et al. A home-based resistance training program for survivors of prostate cancer: A pilot randomized controlled trial. Journal of Science and Medicine in Sport. 2012;15:S333. | Abstract only |
| 50 | Jones LW, Courneya KS, Fairey AS, Mackey JR. Effects of an oncologist's recommendation to exercise on self-reported exercise behavior in newly diagnosed breast cancer survivors: a single-blind, randomized controlled trial. Ann Behav Med. 2004 Oct;28(2):105-13. doi: 10.1207/s15324796abm2802_5. PMID: 15454357. | Wrong patient population |
| 51 | Jones SB, Thomas GA, Hesselsweet SD, Alvarez-Reeves M, Yu H, Irwin ML. Effect of exercise on markers of inflammation in breast cancer survivors: the Yale exercise and survivorship study. Cancer Prev Res (Phila). 2013 Feb;6(2):109-18. doi: 10.1158/1940-6207.CAPR-12-0278. Epub 2012 Dec 4. PMID: 23213072; PMCID: PMC3839104. | Secondary paper |
| 52 | Kiwata JL, Dorff TB, Schroeder ET, Dieli-Conwright CM. Abstract 988: Effect of a supervised exercise intervention on sarcopenic obesity and metabolic syndrome in prostate cancer patients: A randomized pilot study. Cancer Res. 1 July 2017; 77 (13_Supplement): 988. https://doi.org/10.1158/1538-7445.AM2017-988. | Abstract only |
| 53 | Kneis S, Wehrle A, Müller J et al. It’s never too late - balance and endurance training improves functional performance, quality of life, and alleviates neuropathic symptoms in cancer survivors suffering from chemotherapy-induced peripheral neuropathy: results of a randomized controlled trial. BMC Cancer. 2019; 19, 414 . https://doi.org/10.1186/s12885-019-5522-7. | Insufficient recruitment details |
| 54 | Koutoukidis DA, Beeken RJ, Manchanda R, Michalopoulou M, Burnell M, Knobf MT, Lanceley A. Recruitment, adherence, and retention of endometrial cancer survivors in a behavioural lifestyle programme: the Diet and Exercise in Uterine Cancer Survivors (DEUS) parallel randomised pilot trial. BMJ Open. 2017 Oct 8;7(10):e018015. doi: 10.1136/bmjopen-2017-018015. PMID: 28993394; PMCID: PMC5640120. | Wrong intervention |
| 55 | Latka RN, Alvarez-Reeves M, Cadmus L et al. Adherence to a randomized controlled trial of aerobic exercise in breast cancer survivors: the Yale exercise and survivorship study. J Cancer Surviv. 2009; 3, 148–157. https://doi.org/10.1007/s11764-009-0088-z. | Secondary paper |
| 56 | Lin P-J, Heckler CE, Culakova E, Xu H, Dunne RF, Gilmore N, et al. Effects of yoga, cognitive behavioral therapy, and a behavioral placebo on sleep: A nationwide multicenter phase III RCT in cancer survivors. Journal of Clinical Oncology. 2021;39(15_suppl):12017-. | Abstract only |
| 57 | Loh SY, Lee SY, Quek KF, Murray L. Barriers to participation in a randomized controlled trial of Qigong exercises amongst cancer survivors: lessons learnt. Asian Pac J Cancer Prev. 2012;13(12):6337-42. doi: 10.7314/apjcp.2012.13.12.6337. PMID: 23464455. | Secondary paper |
| 58 | Lynch BM, Nguyen NH, Moore MM, Reeves MM, Rosenberg DE, Boyle T, Vallance JK, Milton S, Friedenreich CM, English DR. A randomized controlled trial of a wearable technology-based intervention for increasing moderate to vigorous physical activity and reducing sedentary behavior in breast cancer survivors: The ACTIVATE Trial. Cancer. 2019 Aug 15;125(16):2846-2855. doi: 10.1002/cncr.32143. Epub 2019 Apr 23. PMID: 31012970. | Wrong intervention |
| 59 | Mazzocco K, Masiero M, Mazza M, Radice D, Maisonneuve P, Pravettoni G. Recommendation for “a start to move” program: A 8-week program of incremental physical activity in sedentary breast cancer survivors. Annals of Oncology. 2020; 31:S85. | Abstract only |
| 60 | McGuire R, Examining intervention components for promoting adherence to strength weight training exercise in postmenopausal breast cancer survivors with bone loss, 2008. | Secondary paper |
| 61 | McGuire R, Waltman N, Zimmerman L. Intervention components promoting adherence to strength training exercise in breast cancer survivors with bone loss. West J Nurs Res. 2011 Aug;33(5):671-89. doi: 10.1177/0193945910379004. Epub 2010 Aug 11. PMID: 20702684. | Secondary paper |
| 62 | Milne HM, Wallman KE, Gordon S, Courneya KS. Impact of a combined resistance and aerobic exercise program on motivational variables in breast cancer survivors: a randomized controlled trial. Ann Behav Med. 2008 Oct;36(2):158-66. doi: 10.1007/s12160-008-9059-2. Epub 2008 Sep 16. PMID: 18795388. | Secondary paper |
| 63 | Mustian KM, Lin P-J, Culakova E, Bautista J, Xu H, Mohile SG, Janelsins MC et al. Effects of YOCAS yoga, cognitive behavioral therapy, and survivorship health education on insomnia: A URCC NCORP Research Base Phase III RCT in 740 cancer survivors. 2020; 12005-12005. | Abstract only |
| 64 | Myers JS, Mitchell M, Krigel S, Steinhoff A, Boyce-White A, Van Goethem K, Valla M, Dai J, He J, Liu W, Sereika SM, Bender CM. Qigong intervention for breast cancer survivors with complaints of decreased cognitive function. Support Care Cancer. 2019 Apr;27(4):1395-1403. doi: 10.1007/s00520-018-4430-8. Epub 2018 Aug 21. PMID: 30128855; PMCID: PMC7084167. | Insufficient recruitment details |
| 65 | Naumann F, Munro A, Martin E, Magrani P, Buchan J, Smith C, Piggott B, Philpott M. An individual-based versus group-based exercise and counselling intervention for improving quality of life in breast cancer survivors. A feasibility and efficacy study. Psycho-Oncology. 2012; 21: 1136-1139. https://doi.org/10.1002/pon.2015. | Insufficient recruitment details |
| 66 | Nguyen NH, Vallance JK, Buman MP, Moore MM, Reeves MM, Rosenberg DE, Boyle T, Milton S, Friedenreich CM, English DR, Lynch BM. Effects of a wearable technology-based physical activity intervention on sleep quality in breast cancer survivors: the ACTIVATE Trial. J Cancer Surviv. 2021 Apr;15(2):273-280. doi: 10.1007/s11764-020-00930-7. Epub 2020 Sep 1. PMID: 32875536. | Wrong intervention |
| 67 | Nock NL, Owusu C, Kullman EL, Austin K, Roth B, Cerne S, Harmon C, Moore H, Vargo M, Hergenroeder P, Malone H, Rocco M, Tracy R, Lazarus HM, Kirwan JP, Heyman E, Berger NA. A Community-Based Exercise and Support Group Program in African-American Breast Cancer Survivors (ABCs). J Phys Ther Health Promot. 2013 Dec 1;1(1):15-24. doi: 10.18005/pthp0101003. PMID: 24707505; PMCID: PMC3975605. | Wrong study design |
| 68 | Ott CD, Twiss JJ, Waltman NL, Gross GJ, Lindsey AM. Challenges of recruitment of breast cancer survivors to a randomized clinical trial for osteoporosis prevention. Cancer Nurs. 2006 Jan-Feb;29(1):21-31, quiz 32-3. doi: 10.1097/00002820-200601000-00004. PMID: 16557117. | Wrong intervention |
| 69 | Owusu C, Nock NL, Hergenroeder P, Austin K, Bennet E, Cerne S, Moore H, Petkac J, Schluchter M, Schmitz KH, Webb Hooper M, Atkins L, Asagba O, Wimbley L, Berger NA. IMPROVE, a community-based exercise intervention versus support group to improve functional and health outcomes among older African American and non-Hispanic White breast cancer survivors from diverse socioeconomic backgrounds: Rationale, design and methods. Contemp Clin Trials. 2020 May; 92:106001. doi: 10.1016/j.cct.2020.106001. Epub 2020 Apr 15. PMID: 32304828; PMCID: PMC7325580. | Insufficient recruitment details |
| 70 | Park SH, Knobf MT, Kerstetter J, Jeon S. Adherence to American Cancer Society Guidelines on Nutrition and Physical Activity in Female Cancer Survivors: Results From a Randomized Controlled Trial (Yale Fitness Intervention Trial). Cancer Nurs. 2019 May/Jun;42(3):242-250. doi: 10.1097/NCC.0000000000000602. PMID: 29746264; PMCID: PMC6226367. | Secondary paper |
| 71 | Penttinen H, Nikander R, Blomqvist C, Luoto R, Saarto T. Recruitment of breast cancer survivors into a 12-month supervised exercise intervention is feasible. Contemp Clin Trials. 2009 Sep;30(5):457-63. doi: 10.1016/j.cct.2009.04.007. Epub 2009 Apr 24. PMID: 19394448. | Secondary paper |
| 72 | Peppone LJ, Janelsins MC, Kamen C, Mohile SG, Sprod LK, Gewandter JS, Kirshner JJ, Gaur R, Ruzich J, Esparaz BT, Mustian KM. The effect of YOCAS©® yoga for musculoskeletal symptoms among breast cancer survivors on hormonal therapy. Breast Cancer Res Treat. 2015 Apr;150(3):597-604. doi: 10.1007/s10549-015-3351-1. Epub 2015 Mar 27. PMID: 25814054; PMCID: PMC4467273. | Insufficient recruitment details |
| 73 | Peppone LJ, Janelsins MC, Kamen C, Mohile SG, Sprod LK, Gewandter JS, Kirshner JJ, Gaur R, Ruzich J, Esparaz BT, Mustian KM. The effect of YOCAS©® yoga for musculoskeletal symptoms among breast cancer survivors on hormonal therapy. Breast Cancer Res Treat. 2015 Apr;150(3):597-604. doi: 10.1007/s10549-015-3351-1. Epub 2015 Mar 27. PMID: 25814054; PMCID: PMC4467273. | Duplicate |
| 74 | Peppone LJ, Janelsins MC, Kamen C, Mohile SG, Sprod LK, Gewandter JS, Kirshner JJ, Gaur R, Ruzich J, Esparaz BT, Mustian KM. The effect of YOCAS©® yoga for musculoskeletal symptoms among breast cancer survivors on hormonal therapy. Breast Cancer Res Treat. 2015 Apr;150(3):597-604. doi: 10.1007/s10549-015-3351-1. Epub 2015 Mar 27. PMID: 25814054; PMCID: PMC4467273. | Duplicate |
| 75 | Pinto BM, Trunzo JJ, Rabin C et al. Recruitment Strategies for a Home-Based Physical Activity Intervention for Breast Cancer Patients. Journal of Clinical Psychology in Medical Settings. 2004; 11, 171–178 https://doi.org/10.1023/B:JOCS.0000037611.32348.13 | Secondary paper |
| 76 | Pinto BM, Rabin C, Dunsiger S. Home-based exercise among cancer survivors: adherence and its predictors. Psycho-Oncology. 2009 Apr;18(4):369-76. doi: 10.1002/pon.1465. PMID: 19242921; PMCID: PMC2958525. | Secondary paper |
| 77 | Portela AL, Santaella CL, Gómez CC, Burch A. Feasibility of an Exercise Program for Puerto Rican Women who are Breast Cancer Survivors. Rehabil Oncol. 2008 Apr 1;26(2):20-31. doi: 10.1901/jaba.2008.26-20. PMID: 20664723; PMCID: PMC2910091. | Insufficient recruitment details |
| 78 | Porter LS, Gao X, Lyna P, Kraus W, Olsen M, Patterson E, Puleo B, Pollak KI. Pilot randomized trial of a couple-based physical activity videoconference intervention for sedentary cancer survivors. Health Psychol. 2018 Sep;37(9):861-865. doi: 10.1037/hea0000608. PMID: 30138021; PMCID: PMC9840846. | Wrong intervention |
| 79 | Rogers LQ, Courneya KS, Carter SJ, Anton PM, Verhulst S, Vicari SK, Robbs RS, McAuley E. Effects of a multicomponent physical activity behavior change intervention on breast cancer survivor health status outcomes in a randomized controlled trial. Breast Cancer Res Treat. 2016 Sep;159(2):283-91. doi: 10.1007/s10549-016-3945-2. Epub 2016 Aug 18. PMID: 27539586; PMCID: PMC5014640. | Secondary paper |
| 80 | Schwartz AL, Winters-Stone K. Effects of a 12-month randomized controlled trial of aerobic or resistance exercise during and following cancer treatment in women. Phys Sportsmed. 2009 Oct;37(3):62-7. doi: 10.3810/psm.2009.10.1730. PMID: 20048529. | Primary treatment not completed |
| 81 | Sheehan P, Denieffe S, Harrison M. Evaluation of a Sustainable Intervention using Exercise-for Cancer Fatigue (ESIE-CF Trial).Psycho-Oncology. 2016; 25, 183-184, CN-01759086. https;//10.1002/pon.4272. | Abstract only |
| 82 | Sheehan P, Denieffe S, Murphy NM, Harrison M. Exercise is more effective than health education in reducing fatigue in fatigued cancer survivors. Support Care Cancer. 2020 Oct;28(10):4953-4962. doi: 10.1007/s00520-020-05328-w. Epub 2020 Feb 4. PMID: 32020356. | Wrong study design |
| 83 | Sprod LK, Fernandez ID, Janelsins MC, Peppone LJ, Atkins JN, Giguere J, Block R, Mustian KM. Effects of yoga on cancer-related fatigue and global side-effect burden in older cancer survivors. J Geriatr Oncol. 2015 Jan;6(1):8-14. doi: 10.1016/j.jgo.2014.09.184. Epub 2014 Oct 30. PMID: 25449185; PMCID: PMC4297736. | Secondary paper |
| 84 | Stacey FG, Lubans DR, Chapman K, Bisquera A, James EL. Maintenance of Lifestyle Changes at 12-month Follow-up in a Nutrition and Physical Activity Trial for Cancer Survivors. Am J Health Behav. 2017 Nov 1;41(6):784-795. doi: 10.5993/AJHB.41.6.12. PMID: 29025506. | Secondary paper |
| 85 | Thomas GA, Alvarez-Reeves M, Lu L, Yu H, Irwin ML. Effect of exercise on metabolic syndrome variables in breast cancer survivors. Int J Endocrinol. 2013;2013:168797. doi: 10.1155/2013/168797. Epub 2013 Nov 11. PMID: 24319454; PMCID: PMC3844242. | Secondary paper |
| 86 | Tirado-Gomez M, Palacios C, Ortiz A, Hughes DC, Gonzalez-Mercado V, Vallejo L, Lozada J, Basen-Engquist K. Abstract B32: Serum adipokines and inflammatory markers in Hispanic breast cancer survivors. Cancer Epidemiol Biomarkers. 2015; 24 (10_Supplement): B32. https://doi.org/10.1158/1538-7755.DISP14-B32. | Abstract only |
| 87 | Twiss J, Waltman N, Berg K, Ott C, Lindsey A. An Exercise Intervention for Breast Cancer Survivors With Bone Loss. Journal of nursing scholarship : an official publication of Sigma Theta Tau International Honor Society of Nursing / Sigma Theta Tau. 2009;41:20-7 | Secondary paper |
| 88 | Vallance JK, Courneya KS, Plotnikoff RC, Yasui Y, Mackey JR. Randomized controlled trial of the effects of print materials and step pedometers on physical activity and quality of life in breast cancer survivors. J Clin Oncol. 2007 Jun 10;25(17):2352-9. doi: 10.1200/JCO.2006.07.9988. PMID: 17557948. | Wrong intervention |
| 89 | Vona-Davis L, Abraham J, Bonner D, Gilleland D, Hobbs G, Kurian S, Yanosik MA, Swisher A. Abstract P1-09-12: Effect of a 12-week supervised physical activity and healthy eating program on body weight, functional capacity and serum biomarkers in survivors of triple-negative breast cancer: A randomized, controlled trial. Cancer Res. 1 May 2015; 75 (9_Supplement): P1–09–12. https://doi.org/10.1158/1538-7445.SABCS14-P1-09-12. | Abstract only |
| 90 | Vyas A, Chun Ding Y, Flores S, Johnson M, Riazi G, Mortimer J, Neuhausen S, Clague Dehart J. Abstract 5271: Exercise intervention alleviates inflammation-related biomarkers in breast cancer survivors. Cancer Res. 1 July 2018; 78 (13_Supplement): 5271. https://doi.org/10.1158/1538-7445.AM2018-5271. | Abstract only |
| 91 | Wang LF, Eaglehouse YL, Poppenberg JT, Brufsky JW, Geramita EM, Zhai S, Davis KK, Gibbs BB, Metz J, van Londen GJ. Effects of a personal trainer-led exercise intervention on physical activity, physical function, and quality of life of breast cancer survivors. Breast Cancer. 2021 May;28(3):737-745. doi: 10.1007/s12282-020-01211-y. Epub 2021 Mar 10. PMID: 33689150; PMCID: PMC8386195. | Abstract only |
| 92 | Winger JG, Mosher CE, Rand KL, Morey MC, Snyder DC, Demark-Wahnefried W. Diet and exercise intervention adherence and health-related outcomes among older long-term breast, prostate, and colorectal cancer survivors. Ann Behav Med. 2014 Oct;48(2):235-45. doi: 10.1007/s12160-014-9598-7. PMID: 24648018; PMCID: PMC4156898. | Secondary paper |
| 93 | Wiskemann J, Clauss D, Tjaden C, Hackert T, Schneider L, Ulrich CM, Steindorf K. Progressive Resistance Training to Impact Physical Fitness and Body Weight in Pancreatic Cancer Patients: A Randomized Controlled Trial. Pancreas. 2019 Feb;48(2):257-266. doi: 10.1097/MPA.0000000000001221. PMID: 30589829. | Primary treatment not completed |
| 94 | Yeruva SL, Green D, Martin C, Taylor TR. Factors associated with intention to participate in a randomized yoga intervention among African American breast cancer survivors. Journal of Clinical Oncology. 2016;34(15_suppl):e21538-e. | Abstract only |
